# Supplementary material for: Does workforce explain the relationship between funding and patient experience? A mediation analysis of primary care data in England
Source: BMJ Open. 2024 Feb 19;14(2):e072498. doi: 10.1136/bmjopen-2023-072498 (PMC10882355; doi:10.1136/bmjopen-2023-072498)
Supplement: Supplementary data [file bmjopen-2023-072498supp001.pdf]

Figure 1Sup: Data sample flow chart

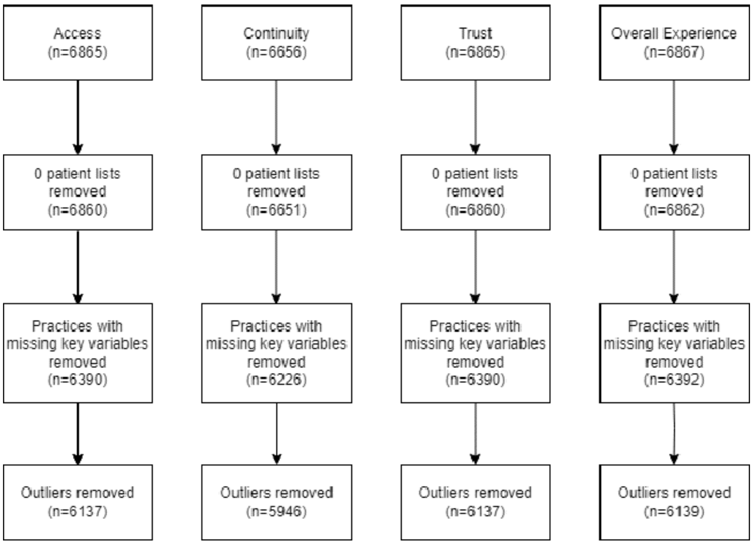

Table 1Sup: Extended practice characteristics for each outcome dataset

	Access	*Continuity*	*Trust*	*Overall Experience*
	Total (N=6137)	Total (N=5946)	Total (N=6137)	Total (N=6139)
Total NHS Payments (£) *Mean (SD)*	1,300,000 (806,000)	1,320,000 (794,000)	1,300,000 (806,000)	1,300,000 (806,000)
Payments per Patient (£) *Mean (SD)*	152 (40.3)	152 (39.4)	152 (40.3)	152 (40.3)
Payments per Weighted Patient (£) *Mean (SD)*	151 (35.4)	151 (34.7)	151 (35.4)	151 (35.4)
Contract Type n (%)				
APMS	136 (2.2%)	125 (2.1%)	136 (2.2%)	136 (2.2%)

GMS	4313 (70.3%)	4176 (70.2%)	4313 (70.3%)	4315 (70.3%)
PMS	1688 (27.5%)	1645 (27.7%)	1688 (27.5%)	1688 (27.5%)
Unknown	0 (0%)	0 (0%)	0 (0%)	0 (0%)
Dispensing n (%)				
Yes	860 (14.0%)	833 (14.0%)	860 (14.0%)	860 (14.0%)
No	5277 (86.0%)	5113 (86.0%)	5277 (86.0%)	5279 (86.0%)
Unknown	0 (0%)	0 (0%)	0 (0%)	0 (0%)
Rurality n (%)				
Rural	919 (15.0%)	885 (14.9%)	919 (15.0%)	919 (15.0%)
Urban	5218 (85.0%)	5061 (85.1%)	5218 (85.0%)	5220 (85.0%)
Registered Patients				
Mean (SD)	8640 (5220)	8790 (5150)	8640 (5220)	8640 (5220)
Weighted Patients				
Mean (SD)	8630 (5120)	8780 (5060)	8630 (5120)	8630 (5120)
FTE GPs per 10,000 patients (pts)				
Mean (SD)	5.61 (2.20)	5.62 (2.19)	5.61 (2.20)	5.61 (2.20)
Total Patient number > 65 yrs				
Mean (SD)	1620 (1210)	1650 (1200)	1620 (1210)	1620 (1210)
% Patients with long-standing health condition				
Mean (SD)	51.3 (8.58)	51.3 (8.58)	51.3 (8.58)	51.3 (8.59)

Patient Experience rating (%)<br>*Mean (SD)*<br>*[Min, Max]*	69.1 (14.4)<br>[19.1, 100]	49.1 (18.5)<br>[2.14, 98.0]	95.3 (3.80)<br>[71.6, 100]	83.4 (9.74)<br>[32.2, 100]
Response Rate (%)<br>*Mean (SD)*	35.9 (10.8)	36.0 (10.8)	35.9 (10.8)	35.8 (10.8)

Table 2Sup: Access, Continuity and Trust mediation models

	Access		Continuity		Trust	
	Coefficient	CI: 95%	Coefficient	CI: 95%	Coefficient	CI: 95%
Unadjusted Model						
**Direct Effect of predictor on outcome**	0.044	(0.037; 0.06)	0.044	(0.030; 0.06)	0.007	(0.006; 0.01)
**Total Effect of predictor on outcome**	0.062	(0.053; 0.07)	0.050	(0.036; 0.06)	0.013	(0.010; 0.01)
**Indirect (Mediated) Effect**	0.016	(0.013; 0.02)	0.006	(0.004; 0.01)	0.005	(0.004; 0.01)
Carr-Hill adjusted Model						
**Direct Effect of predictor on outcome**	0.048	(0.037; 0.06)	0.037	(0.022; 0.06)	0.007	(0.004; 0.01)

**Total Effect of predictor on outcome**	0.060	(0.048; 0.07)	0.041	(0.026; 0.06)	0.010	(0.0073; 0.01)
**Indirect (Mediated) Effect**	0.011	(0.009; 0.01)	0.004	(0.002; 0.01)	0.003	(0.0025; 0.0035)
Deprivation adjusted Model<br>**Direct Effect of predictor on outcome**	0.047	(0.038; 0.06)	0.045	(0.030; 0.06)	0.008	(0.006; 0.010)
**Total Effect of predictor on outcome**	0.061	(0.053; 0.07)	0.050	(0.035; 0.06)	0.012	(0.010; 0.014)
**Indirect (Mediated) Effect**	0.015	(0.012; 0.02)	0.005	(0.003; 0.01)	0.004	(0.003; 0.005)
