# Supplementary material for: Does workforce explain the relationship between funding and patient experience? A mediation analysis of primary care data in England
Source: BMJ Open. 2024 Feb 19;14(2):e072498. doi: 10.1136/bmjopen-2023-072498 (PMC10882355; doi:10.1136/bmjopen-2023-072498)
Supplement: Author's manuscript [file bmjopen-2023-072498.draft_revisions.pdf]

BMJ Open is committed to open peer review. As part of this commitment we make the peer review history of every article we publish publicly available.

When an article is published we post the peer reviewers' comments and the authors' responses online. We also post the versions of the paper that were used during peer review. These are the versions that the peer review comments apply to.

The versions of the paper that follow are the versions that were submitted during the peer review process. They are not the versions of record or the final published versions. They should not be cited or distributed as the published version of this manuscript.

BMJ Open is an open access journal and the full, final, typeset and author-corrected version of record of the manuscript is available on our site with no access controls, subscription charges or pay-per-view fees (http://bmjopen.bmj.com).

If you have any questions on BMJ Open's open peer review process please email info.bmjopen@bmj.com

BMJ Open

Inequalities in primary care: an exploration of the causal pathways linking practice funding, workforce and patient experience

Journal:	*BMJ Open*
Manuscript ID	bmjopen-2023-072498
Article Type:	Original research
Date Submitted by the Author:	07-Feb-2023

Complete List of Authors:	Salant, Natasha; University of Cambridge, Public Health and Primary Care<br>Massou , Efthalia ; University of Cambridge, Public Health and Primary Care<br>Awan, Hassan; Keele University<br>Ford, John; University of Cambridge, Public Health

Keywords:	Primary Health Care, STATISTICS & RESEARCH METHODS, Quality in health care < HEALTH SERVICES ADMINISTRATION & MANAGEMENT

SCHOLARONE™
Manuscripts

I, the Submitting Author has the right to grant and does grant on behalf of all authors of the Work (as defined in the below author licence), an exclusive licence and/or a non-exclusive licence for contributions from authors who are: i) UK Crown employees; ii) where BMJ has agreed a CC-BY licence shall apply, and/or iii) in accordance with the terms applicable for US Federal Government officers or employees acting as part of their official duties; on a worldwide, perpetual, irrevocable, royalty-free basis to BMJ Publishing Group Ltd ("BMJ") its licensees and where the relevant Journal is co-owned by BMJ to the co-owners of the Journal, to publish the Work in this journal and any other BMJ products and to exploit all rights, as set out in our licence.

The Submitting Author accepts and understands that any supply made under these terms is made by BMJ to the Submitting Author unless you are acting as an employee on behalf of your employer or a postgraduate student of an affiliated institution which is paying any applicable article publishing charge ("APC") for Open Access articles. Where the Submitting Author wishes to make the Work available on an Open Access basis (and intends to pay the relevant APC), the terms of reuse of such Open Access shall be governed by a Creative Commons licence – details of these licences and which Creative Commons licence will apply to this Work are set out in our licence referred to above.

Other than as permitted in any relevant BMJ Author's Self Archiving Policies, I confirm this Work has not been accepted for publication elsewhere, is not being considered for publication elsewhere and does not duplicate material already published. I confirm all authors consent to publication of this Work and authorise the granting of this licence.

For peer review only

Inequalities in primary care: an exploration of the causal pathways linking practice funding, workforce and patient experience.

Natasha Salant[1]

Efthalia (Lina) Massou[1]

Hassan Awan[2]

John Ford[1]

[1] University of Cambridge

[2] University of Keele

Corresponding author: Natasha Salant, University of Cambridge MPhil Population Health Sciences 2021-2022, natashasalant@gmail.com / nls40@cantab.ac.uk

For peer review only

ABSTRACT

Objectives: To determine whether GP supply explains part of the mechanism through which practice funding affects patient experience at the level of general practice in England, and if it does, whether the chain of effects is patterned by socio-economic deprivation.

Setting: Primary care practice level analysis of workforce, funding and patient experience of all NHS practices in England.

Primary and secondary outcome measures: A mediation analysis tested the significance of the mediation of GPs per 10,000 patients on the effect of payments per patient, on patient experience. Non-parametric bootstrapping was used to estimate the average causal mediation effect and confidence intervals. Unadjusted models were built and tested before testing models with added covariates.

Participants: The study considered all general practices in England for which there was relevant data for each primary variable. Practices were excluded where patient list size was 0, and where the workforce variable (staff per 10,000 patients) was recorded as being more than 3 SD from the mean. After exclusion, 6139 practices were included in the final analysis.

Results: We found that GPs statistically significantly ($p<0.001$) mediate the effect of practice funding on overall patient experience even when adjusting for rurality, sex and age, and deprivation. The mediated effect constitutes on average 30% of the total effect of practice funding on patient experience.

Conclusions: This study adds to the growing body of evidence on the relationships between workforce, practice funding and quality of care; and moreover by exploring the mechanism by which practice funding affects patient experience, the results are informative for policy strategies to reduce inequality of practice funding, patient experience and workforce supply.

STRENGTHS AND LIMITATIONS

- This study is the first to explore the association between practice-level NHS payments per patient, patient experience and workforce through a mediation analysis. It therefore strengthens the body of knowledge on these three variables by examining the mechanism by which they are associated.
- Given that publicly available datasets are used for this analysis, the data quality of the analysis suffers the same quality issues as these datasets. For instance, there is known inflation of full-time equivalent figures in the NHS Workforce dataset.
- The analysis is limited to NHS payments to practice and therefore does not capture PCN and CCG payments as well as payments for supplementary activities.
- There is a question of endogeneity with regard to whether workforce supply may be due to factors correlated with payments to practice or patient satisfaction. The analysis attempts to control for these factors in the model.

For peer review only

For peer review only

INTRODUCTION

Patient experience is an important measure of primary health quality because it provides information on the process of care that cannot be replaced by other indicators (35), for instance waiting times and interaction with staff. Moreover, it has been linked to health outcomes insofar as whether or not a patient sees their preferred doctor, continuity of care has been linked to cost-effectiveness, reduced emergency hospital admissions, and reduced mortality (36). Historically, patient satisfaction of general practice in the UK has been high; but it has been in decline since 2015 and the gap between socio-economic groups have been widening (1,2). On most measures of quality, practices in more deprived areas fare worse: they have lower quality scores, as measured by the Quality Outcomes Framework, and are more likely to be rated as 'inadequate' by the Care Quality Commission (1).

Widening inequalities in patient satisfaction has coincided with a widening of inequalities in the distribution of the primary care workforce. The distribution of general practitioners (GPs), paramedics, and other allied health professionals favours more affluent areas (1,3,4)(1,5–8). GP density has been found to be associated with higher self-reported quality of care measures (9) and both GP and patient satisfaction (10). While previous research did not find a significant association between GP supply and emergency admissions, there was an association more deprived areas (11).

Some have suggested that funding inequalities are a key driving factor behind inequalities in workforce and satisfaction(1). Practices in more deprived areas receive 7% less general practice funding (1). Capitation funding for general practice is positively associated with overall practice quality and Care Quality Commission ratings (12). In addition, there is evidence of an association between funding and patient experience, whereby practices that receive less funding have lower levels of satisfaction (2). However, as it stands, there has been no robust investigation of the mechanisms of this relationship; in other words, the question of *how* the variables interact remains unknown.

The aim of this research is to explore whether the relationship between funding and patient experience is mediated by the number of GPs, and if a significant mediation effect is found, whether the effect remains the same when adjusting for patient need and deprivation.

For peer review only

METHODS

Data Sources

Four publicly available datasets were used: 'NHS Payments to General Practice' (13), 'General Practice Workforce' (14), GP Patient Survey Data (15), and 'National General Practice Profiles' (16).

NHS Payments data consisted of payments to individual general practice providers in England. Total NHS payments made to general practices included the core capitation amount (global sum) and other payment categories including Quality Outcomes Framework (QoF) payments and payments for premises. The dataset included list numbers of registered and weighted patients. Weighted patient lists were calculated using the Carr-Hill formula (17).

General Practice Workforce data are uploaded by practices every quarter and covers all general practices in England. GP data are available for Full Time Equivalent (FTE) across all partners, salaried GPs, training grades and locums.

The GP Patient Survey (GPPS) is a national self-reported patient experience survey of over 900,000 adults per year in England (18). The results are weighted by age, sex, region and socioeconomic status to ensure representativeness (2). Survey results provided practice-level data that is comparable across practices and time (19).

National General Practice Profiles are a set of approximately 150 general practice level indicators that are produced by the Office for Health Improvement and Disparities for practices across England. The Index of Multiple Deprivation (IMD) score is an area-based measure of socio-economic deprivation that comprises of seven distinct weighted domains: Income (22.5%), Employment (22.5%), Health Deprivation and Disability (13.5%), Education, Skills and Training (13.5%), Crime (9.3%), Barriers to Housing and Services (9.3%), and Living Environment (9.3%) (20).

Variables

Predictor

The predictor variable for practice funding was obtained by dividing 'total NHS payment' by 'registered patient' or 'weighted patient' to create a payment per registered patient or per weighted patient.

Outcome

Four patient experience variables were used from the GP Patient Survey based on previous studies(2,21).

(a) proportion of respondents who had a good or very good experience of making an appointment (Access),
(b) proportion of respondents who always see their preferred GP (Continuity),
(c) proportion of respondents who had trust and confidence in their GP (Trust), and
(d) proportion of respondents who had an overall good or very good experience (Overall Experience).

Mediator

Full time equivalent (FTE) GPs per 10,000 registered or weighted patients were the mediator based on the workforce dataset.

For peer review only

Covariates

An adjusted model was undertaken using patient weights and covariates. Weighted payments per patient takes into account patient sex, age, long-standing health condition and rurality, so these were not added as separate variables. IMD was also added as a covariate.

Sample

We included all general practices in England with data available. Where practices were entirely missing from payments, workforce and patient experience datasets, they were excluded. In addition, practices were excluded if the patient list size was 0 or where workforce data were improbable (defined as more the 3 standard deviations from the mean).

Data merge

Relevant variables from data sources were linked using practice code, and sense-checked by scanning that practice names matched. To account for the lag between funding, employment and patient experience, 2017/18funding data were used, December 2018 workforce data and March 2019 patient experience data. Pandemic data were not used because of data quality issues and challenges around accounting for the impact of changes to services.

Statistical analysis

Three mediation model structures were built using the Baron and Kenny approach (22) also known as the causal steps approach, and all models assumed the causal structure in Figures 1 and 2. These were: an unadjusted model (where GPs per registered patient was tested as a mediator of the effect of payments per registered patient on patient experience), a weighted patients' model (where GPs per weighted patient was tested as a mediator of payments per weighted patient on patient experience) and a deprivation adjusted model (where the unadjusted model was adjusted for deprivation). The weighted model was weighted using the Carr-Hill formula which takes into account, patient sex, age, long-standing health condition and rurality. For each model structure, there were four distinct models that tested each distinct patient experience variable.

In total, twelve mediation models were tested. For each, a simple linear regression of the predictor on the outcome was run to test whether there was a significant association. Mediation analysis was considered if the outcome variable was significantly associated with the predictor variable, the mediator variable was significantly associated with the predictor variable, and controlling for the predictor variable, that the mediator variable associated with the outcome variable. The significance of indirect effect was tested using bootstrapping procedures. Unstandardised indirect effects were computed for each of 1000 bootstrapped samples, and the 95% confidence interval was computed by determining the indirect effects at the 2.5th and 97.5th percentiles. If mediation was established using simple linear regressions, the model was adjusted for patient sex, age, long-standing health condition and rurality.

In addition, the model's sensitivity to removing outliers was tested by running the same analyses on datasets that did not remove outliers.

All analyses were conducted using R (version 4.2) and RStudio.

For peer review only

Patient and Public Involvement

No patient involved.

For peer review only

RESULTS

The final number of practices included in each outcome's dataset varied from 5,946 to 6,139 (Table 1). The continuity dataset included the fewest practices because the survey's continuity question was answered by 2% fewer participants than access, trust and overall experience questions. Across all datasets, the average practice receives NHS payments of £152 per registered patient and the average practice size is 8,640 patients. Most practices have a General Medical Services (GMS) contract, do not dispense and are based in urban areas. Practices employ on average 5.6 FTE GPs. The average practice receives a good or very good rating of 83% for overall patient experience, 69% for access, 49% for continuity and 95% for trust.

Table 1: Practice characteristics for each outcome dataset

	Access	Continuity	Trust	Overall Experience
	Total (N=6137)	Total (N=5946)	Total (N=6137)	Total (N=6139)
Total NHS Payments (£) Mean (SD)	1,300,000 (806,000)	1,320,000 (794,000)	1,300,000 (806,000)	1,300,000 (806,000)
Payments per Patient (£) Mean (SD)	152 (40.3)	152 (39.4)	152 (40.3)	152 (40.3)
Payments per Weighted Patient (£) Mean (SD)	151 (35.4)	151 (34.7)	151 (35.4)	151 (35.4)
Contract Type n (%)				
APMS	136 (2.2%)	125 (2.1%)	136 (2.2%)	136 (2.2%)
GMS	4313 (70.3%)	4176 (70.2%)	4313 (70.3%)	4315 (70.3%)
PMS	1688 (27.5%)	1645 (27.7%)	1688 (27.5%)	1688 (27.5%)
Unknown	0 (0%)	0 (0%)	0 (0%)	0 (0%)
Dispensing n (%)				
Yes	860 (14.0%)	833 (14.0%)	860 (14.0%)	860 (14.0%)
No	5277 (86.0%)	5113 (86.0%)	5277 (86.0%)	5279 (86.0%)
Unknown	0 (0%)	0 (0%)	0 (0%)	0 (0%)
Rurality n (%)				
Rural	919 (15.0%)	885 (14.9%)	919 (15.0%)	919 (15.0%)
Urban	5218 (85.0%)	5061 (85.1%)	5218 (85.0%)	5220 (85.0%)
Registered Patients Mean (SD)	8640 (5220)	8790 (5150)	8640 (5220)	8640 (5220)
Weighted Patients Mean (SD)	8630 (5120)	8780 (5060)	8630 (5120)	8630 (5120)
FTE GPs per 10,000 patients (pts) Mean (SD)	5.61 (2.20)	5.62 (2.19)	5.61 (2.20)	5.61 (2.20)

Total Patient number > 65 yrs Mean (SD)	1620 (1210)	1650 (1200)	1620 (1210)	1620 (1210)
% Patients with long-standing health condition Mean (SD)	51.3 (8.58)	51.3 (8.58)	51.3 (8.58)	51.3 (8.59)
Patient Experience rating (%) Mean (SD) [Min, Max]	69.1 (14.4) [19.1, 100]	49.1 (18.5) [2.14, 98.0]	95.3 (3.80) [71.6, 100]	83.4 (9.74) [32.2, 100]
Response Rate (%) Mean (SD)	35.9 (10.8)	36.0 (10.8)	35.9 (10.8)	35.8 (10.8)

When testing an unadjusted mediation model, the effect of payments per registered patient on overall patient experience was partially mediated via GPs per 10,000 patients (Figure 3). The regression coefficient between payments per patient and overall patient experience (0.030) and the regression coefficient between GPs per 10,000 patients and overall patient experience (1.213) were statistically significant (p<0.001). The total effect of payments per patient on overall patient experience was 0.044. The indirect effect was 0.014 (95%CI 0.011 to 0.017). The model could be seen to illustrate that a £10 increase in practice funding is associated with a 0.44% increase in overall patient experience. The proportion of the effect of payments per registered patient on overall patient experience that is mediated by GPs per 10,000 is 32%; or in other words, the increase in payments per patient that drives an increase in GPs per 10,000 patients explains more than one third of the effect on an increase in overall patient experience.

Figure 4 shows the effect of payments per weighted patient on overall patient experience was partially mediated via GPs per 10,000 weighted patients. The regression coefficient between payments per weighted patient and overall patient experience (0.028) and the regression coefficient between GPs per 10,000 patients and overall patient experience (1.152) were significant (p<0.001), and very similar to the unadjusted model. The indirect effect was 0.010 (95%CI 0.008 to 0.012). This weighted model could be seen to illustrate that a £10 increase in practice funding is associated with a 0.38% increase in overall patient experience, and the proportion of the total effect that payments per weighted patient has on overall patient experience is 26%. This reflects the finding that weighted payments per patient have a marginally smaller effect (0.038, p<0.001) on overall patient experience.

When adjusted for socio-economic deprivation, the effect of payments per patient on overall patient experience is still significantly (p<0.001) partially mediated by GPs per 10,000 patients (Figure 5). In this adjusted model, the indirect effect was 0.013 (95% CI, 0.011 to 0.015). The total effect of payments per registered patient on overall patient experience (0.043) has the same effect size as the unadjusted model and is still significant (p<0.001). However, the direct effect (0.030) and indirect effect (0.013), while still both significant (p<0.001), differ slightly in size from the simple model, and the proportion mediated is 30%.

Mediation models with access, trust and continuity indicators as outcome variables were all similarly significantly mediated (p<0.001) by GPs per 10,000 patients. Coefficients and confidence intervals for these are detailed in Table 2.

Table 2: Access, Continuity and Trust mediation models

	Access		Continuity		Trust	
	Coefficient	CI: 95%	Coefficient	CI: 95%	Coefficient	CI: 95%
**Unadjusted Model**						
Direct Effect of predictor on outcome	0.044	(0.037; 0.06)	0.044	(0.030; 0.06)	0.007	(0.006; 0.01)
Total Effect of predictor on outcome	0.062	(0.053; 0.07)	0.050	(0.036; 0.06)	0.013	(0.010; 0.01)
Indirect (Mediated) Effect	0.016	(0.013; 0.02)	0.006	(0.004; 0.01)	0.005	(0.004; 0.01)
**Carr-Hill adjusted Model**						
Direct Effect of predictor on outcome	0.048	(0.037; 0.06)	0.037	(0.022; 0.06)	0.007	(0.004; 0.01)
Total Effect of predictor on outcome	0.060	(0.048; 0.07)	0.041	(0.026; 0.06)	0.010	(0.0073; 0.01)
Indirect (Mediated) Effect	0.011	(0.009; 0.01)	0.004	(0.002; 0.01)	0.003	(0.0025; 0.0035)
**Deprivation adjusted Model**						
Direct Effect of predictor on outcome	0.047	(0.038; 0.06)	0.045	(0.030; 0.06)	0.008	(0.006; 0.010)
Total Effect of predictor on outcome	0.061	(0.053; 0.07)	0.050	(0.035; 0.06)	0.012	(0.010; 0.014)
Indirect (Mediated) Effect	0.015	(0.012; 0.02)	0.005	(0.003; 0.01)	0.004	(0.003; 0.005)

For peer review only

All access models had the highest association coefficients between practice funding and patient experience, with the unadjusted model demonstrating that a £10 pound increase in practice funding was associated with a 0.44% increase in patient experience, of which 36% could be explained by an increase of 1 GP per 10,000 patients. When adjusting for workload weighting and deprivation, the effect size remained the highest out of all models, with GP workforce accounting for ~31% of the effect size. Direct, total and mediated effect sizes were substantially smaller for the continuity and trust models indicating that while an increase in GPs per 10,000 patients was a statistically significant mediator for trust and continuity measures of patient experience, the effect may be more relevant for access and overall experience measures.

Sensitivity analyses including the data outliers did not have a substantial impact on the results.

For peer review only

DISCUSSION

Main finding of this study

We found that the number of GPs per 10,000 patients statistically significantly mediates the effect of practice funding on overall patient experience, access, trust and continuity. In addition, the mediation effect was statistically significant when adjusting for workload, rurality and staff market forces, as well as deprivation.

While all relationships in the primary mediation analysis were found to be significant, the effect sizes were small. In the unadjusted model, the total effect size of the association between payments per patient and overall patient experience was 0.044, which can be interpreted to mean that an increase of £10 in payments per patient is associated with an increase of 0.4%in overall patient experience. Given that the mean payment per patient is £152, this means that for the average practice, even with a funding increase of 6%, overall patient experience score would only increase marginally. Nevertheless, the results suggest that 30% of the explanation for an increase in payments per patient being associated with an increase in overall patient experience is the increase in FTE GPs per 10,000 patients. This finding is important because it suggests that increasing funding may only have a small impact on patient experience and only 30% of any improvements would by employing more staff.

Strengths and limitations

The study designed was strengthened by building up the mediation model step by step, allowing an understanding of the associations at each level of the mediation. In addition, it uses a non-parametric estimation of the indirect effect and significance, which, because of its wide applicability to a variety of models, resolves any doubts arising from whether or not the method of estimation and significance testing is suitable for this model (23,24).

GP Patient Survey response rate is about 30%, raising questions about whether the data is suitable for a quantitative England-wide study. However, because responses are weighted to account for selection bias, demographic characteristics of the eligible population, as well as differences between responders and non-responders (25), this problem is mitigated. In addition, both the General Practice Workforce and NHS Payments datasets have known quality considerations. For the Workforce dataset, there is known inflation of FTE and headcount figures due to the recruitment of temporary staff to cover staff on long-term leave (26). For the NHS Payments dataset, patient list numbers should be viewed with caution given that there is known list inflation whereby patients who have left a practice are not deregistered (13).

This analysis of practice funding is limited to an analysis of NHS payments to practice and does not include practice funding received from other sources such as research or training. However, this limitation addresses a wider problem that practice-level data on additional funding, expenditure and profit data is not publicly available.

What this study adds

This study is the first to explore the association between practice-level NHS payments per patient, patient experience and workforce through mediation analyses. Despite prior evidence to show that an increase in NHS practice funding is associated with an increase in patient experience (2) and CQC

For peer review only

scores (12), it is unclear from these studies how increasing practice funding may lead to improved quality outcomes. Understanding the mechanisms of action is valuable to recognise correlates of increasing GP per patient ratios, identify practices that may be more or less likely to benefit from an increase in payments per patient, or inform policy so that increasing practice funding can be more strategic. Payments per weighted patient are not equal per decile, with more affluent deciles receiving more funding per weighted patient. That information is enriched by the finding that the inequality in payments is associated with an inequality in capability to recruit GPs, which plays a substantial role in patient experience.

Moreover, this study read together with previous analyses on the widening inequality GP distribution (7,8) should be interpreted to mean that if the status quo continues with regard to NHS payments to practices, i.e. a total increase in average payments per patient of approximately 4% over the past four years (27), the inequality in payments per weighted patient, as well as that in GPs per 10,000 weighted patients, will likely continue to grow. Given the results of this study, it is unsurprising that recent results from the GP Patient Survey reveal a widening gap in patient experience, with patients attending practices in more deprived neighbourhoods reporting worse overall experience than patients attending practices in more affluent neighbourhoods (28).

Investment in general practice in England is thought to have lagged behind what is necessary to finance increasing total expenditure, population growth, increasing consultations and expanding complexity of health needs (29). While undeniable value for money can be achieved with primary care investment (30,31), the results of this study do not suggest that simply investing more money in practices would solve the problem. While practice payments per patient significantly impact the number of GPs per 10,000 patients, the effect size is small. This reflects that workforce recruitment is a problem that is not entirely dependent on practice funding. However, if the system is committed to improving quality, policy must target both practice funding, GP recruitment and quality improvement.

Policy recommendations

Review of the contractual funding model (Carr-Hill formula) should be reviewed to address the unequal distribution of payments per weighted patient. Its inaccurate reflection of patient need is identified in the inequality of average practice payments per weighted patients, and according to this analysis this has an impact on the distribution of GPs per 10,000 patients as well as on inequality of patient experience. In addition, given the effect of practice funding on GPs per 10,000 patients identified by this study, as well as the widening inequality of GP distribution found in multiple previous studies, it is recommended that additional funding is identified and targeted towards underdoctored areas to stop the inequality from increasing. This targeted investment could have a knock-on effect on patient experience in these areas, according to the findings of this study. Finally, given the demonstrable link between funding, GP supply and patient experience, it is recommended that practices achieving low quality of care scores are not punished through missing out on financial incentives; but rather practices are supported to improve quality of care.

CONCLUSION

We found that the number of FTE GPs per 10,000 patients is a significant mediator, and explains a third of the mechanism of the effect of payments per patient on patient experience. This is particularly important in light of the additional findings that the distribution of GPs per 10,000

For peer review only

weighted patients is unequal across deciles, with on average higher ratios of GPs to patients weighted for need in practices serving more affluent populations; and that when payments are weighted for patient need, more affluent practice populations benefit from more NHS practice funding on average. Targeted policy action is required urgently to address issues of resource allocation, inequality of GP recruitment and retention, and quality of care.

Figure Legend

Figure 1: Assumed causal structure of unadjusted mediation model

Figure 2: Assumed causal structure of adjusted mediation model

Figure 3: Unadjusted mediation model results

Figure 4: Deprivation adjusted mediation model results

Figure 5: Carr-Hill adjusted mediation model results

Contributorship statement

Natasha Salant is the primary author having made substantial contributions to the conception and design of the work, the acquisition of the data, the cleaning and analysis of the data as well as the interpretation thereof. She is also responsible for drafting of the work, revising it, and final approval for it to be published, thereby agreeing to be accountable for all aspects of the work. Hassan Awan made substantial contributions to the interpretation of data, the critical revision and final approval for publication thereby agreeing to be accountable for the work. Efthalia Massou made substantial contributions to the design of the statistical analysis as well as the interpretation of the analysis, critical revision of the draft, and final approval for publication thereby being accountable for the work. John Ford made substantial contributions to the concept and design of the work as well as to the interpretation of the data, critical revision of the draft, and final approval for publication thereby agreeing to be accountable for the work.

Competing Interests

No authors have any conflicting interests to declare.

For peer review only

Funding

Natasha Salant is grateful to Chevening Fellowships, the UK government's global scholarship programme, funded by the Foreign, Commonwealth and Development Office (FCDO) and partner organisations for the funding of her MPhil program, thereby making this research possible.

Data sharing statement

Datasets used are available publicly as per references provided for each dataset used. Code for analysis is available upon request.

Ethics Approval

This study does not involve human participants and ethical approval was not required.

For peer review only

References

1. Level or not? - The Health Foundation [Internet]. [cited 2022 Jan 1]. Available from: https://www.health.org.uk/publications/reports/level-or-not

2. L'Esperance V, Gravelle H, Schofield P, Ashworth M. Impact of primary care funding on patient satisfaction: a retrospective longitudinal study of English general practice, 2013–2016. Br J Gen Pract. 2020 Dec 1;71(702):e47–54.

3. The bigger picture - The Health Foundation [Internet]. [cited 2022 Jul 16]. Available from: https://www.health.org.uk/publications/reports/the-bigger-picture

4. Tackling the inverse care law - The Health Foundation [Internet]. [cited 2022 Jul 8]. Available from: https://www.health.org.uk/publications/reports/tackling-the-inverse-care-law

5. 'Levelling up' general practice in England - The Health Foundation [Internet]. [cited 2022 Jun 24]. Available from: https://www.health.org.uk/publications/long-reads/levelling-up-general-practice-in-england

6. Cookson R, Propper C, Asaria M, Raine R. Socio-Economic Inequalities in Health Care in England. Fisc Stud. 2016;37(3–4):371–403.

7. Nussbaum C, Massou E, Fisher R, Morciano M, Harmer R, Ford J. Inequalities in the distribution of the general practice workforce in England: a practice-level longitudinal analysis. BJGP Open [Internet]. 2021 Oct 1 [cited 2022 Apr 12];5(5). Available from: https://bjgpopen.org/content/5/5/BJGPO.2021.0066

8. Asaria M, Cookson R, Fleetcroft R, Ali S. Unequal socioeconomic distribution of the primary care workforce: whole-population small area longitudinal study. BMJ Open. 2016 Jan 19;6(1):e008783.

9. Vallejo-Torres L, Morris S. Primary care supply and quality of care in England. Eur J Health Econ. 2018;19(4):499–519.

10. Gibson J, Francetic I, Spooner S, Checkland K, Sutton M. Primary care workforce composition and population, professional, and system outcomes: a retrospective cross-sectional analysis. Br J Gen Pract. 2022 May 1;72(718):e307–15.

11. Nicodemo C, McCormick B, Wittenberg R, Hobbs FR. Are more GPs associated with a reduction in emergency hospital admissions? A quantitative study on GP referral in England. Br J Gen Pract. 2021 Mar 9;71(705):e287–95.

12. L'Esperance V, Gravelle H, Schofield P, Santos R, Ashworth M. Relationship between general practice capitation funding and the quality of primary care in England: a cross-sectional, 3-year study. BMJ Open. 2019 Nov 7;9(11):e030624.

13. NHS Payments to General Practice - NHS Digital [Internet]. [cited 2022 Jan 31]. Available from: https://digital.nhs.uk/data-and-information/publications/statistical/nhs-payments-to-general-practice

14. General Practice Workforce [Internet]. NHS Digital. [cited 2022 Jan 31]. Available from: https://digital.nhs.uk/data-and-information/publications/statistical/general-and-personal-medical-services

For peer review only

15. Survey and Reports [Internet]. [cited 2022 Feb 3]. Available from: https://www.gp-patient.co.uk/surveysandreports

16. National General Practice Profiles - OHID [Internet]. [cited 2022 Feb 5]. Available from: https://fingertips.phe.org.uk/profile/general-practice

17. Dyson M. Global sum allocation formula [Internet]. The British Medical Association is the trade union and professional body for doctors in the UK. [cited 2021 Dec 8]. Available from: https://www.bma.org.uk/advice-and-support/gp-practices/funding-and-contracts/global-sum-allocation-formula

18. GP Patient Survey – About [Internet]. [cited 2022 Feb 3]. Available from: https://www.gp-patient.co.uk/About

19. Tallett A, Poots AJ, Graham C, Peters M, Corbett R, Sizmur S, et al. How can patient experience scores be used to predict quality inspection ratings? A retrospective cross-sectional study of national primary care datasets in the UK. BMJ Open. 2020 Nov 26;10(11):e041709.

20. English indices of deprivation [Internet]. GOV.UK. [cited 2022 Feb 9]. Available from: https://www.gov.uk/government/collections/english-indices-of-deprivation

21. Saunders CL, Flynn S, Massou E, Lyratzopoulos G, Abel G, Burt J. Sociodemographic inequalities in patients' experiences of primary care: an analysis of the General Practice Patient Survey in England between 2011 and 2017. J Health Serv Res Policy. 2021 Jul 1;26(3):198–207.

22. Baron RM, Kenny DA. The moderator–mediator variable distinction in social psychological research: Conceptual, strategic, and statistical considerations. J Pers Soc Psychol. 1986 Dec;51(6):1173–82.

23. Imai K, Keele L, Yamamoto T. Identification, Inference and Sensitivity Analysis for Causal Mediation Effects. Stat Sci [Internet]. 2010 Feb 1 [cited 2022 Jul 13];25(1). Available from: https://projecteuclid.org/journals/statistical-science/volume-25/issue-1/Identification-Inference-and-Sensitivity-Analysis-for-Causal-Mediation-Effects/10.1214/10-STS321.full

24. Imai K, Keele L, Tingley D. A general approach to causal mediation analysis. Psychol Methods. 2010 Dec;15(4):309–34.

25. GPPS Technical Annex 2019 [Internet]. [cited 2022 Jul 14]. Available from: https://www.gp-patient.co.uk/downloads/2019/GPPS_2019_Technical_Annex_PUBLIC.pdf

26. Background Data Quality Statement [Internet]. NHS Digital. [cited 2022 Jul 15]. Available from: https://digital.nhs.uk/data-and-information/publications/statistical/general-and-personal-medical-services/31-october-2021/background-data-quality-statement

27. Executive Summary [Internet]. NHS Digital. [cited 2022 Jul 15]. Available from: https://digital.nhs.uk/data-and-information/publications/statistical/nhs-payments-to-general-practice/england-2020-21/executive-summary-copy

28. Statistics » GP Patient Survey 2022 [Internet]. [cited 2022 Jul 15]. Available from: https://www.england.nhs.uk/statistics/2022/07/14/gp-patient-survey-2022/#

29. Dyson M. Investment in general practice [Internet]. The British Medical Association is the trade union and professional body for doctors in the UK. [cited 2022 Jul 27]. Available from:

For peer review only

https://www.bma.org.uk/advice-and-support/nhs-delivery-and-workforce/funding/investment-in-general-practice

30. Starfield B, Shi L, Macinko J. Contribution of Primary Care to Health Systems and Health. Milbank Q. 2005;83(3):457–502.

31. Schäfer WLA, Boerma WGW, van den Berg MJ, De Maeseneer J, De Rosis S, Detollenaere J, et al. Are people’s health care needs better met when primary care is strong? A synthesis of the results of the QUALICOPC study in 34 countries. Prim Health Care Res Dev. 2019 Jul 1;20:e104.

For peer review only

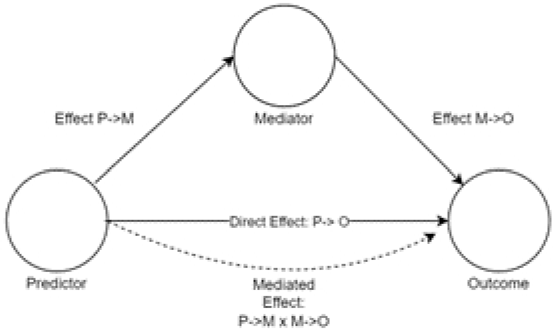

Figure 1

191x118mm (38 x 38 DPI)

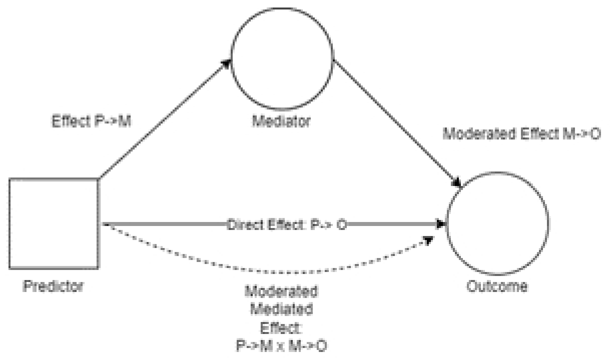

Figure 2

207x118mm (38 x 38 DPI)

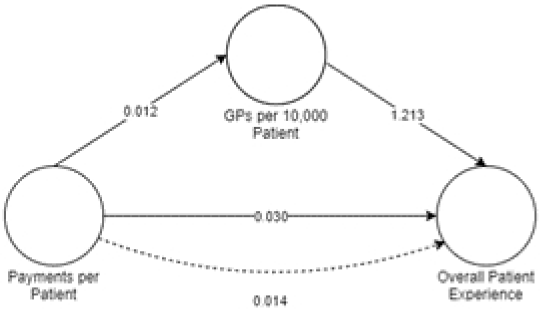

Figure 3

181x101mm (38 x 38 DPI)

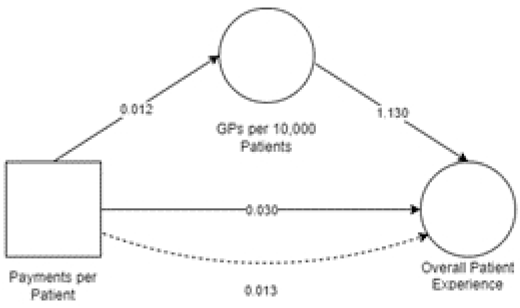

Figure 4

174x102mm (38 x 38 DPI)

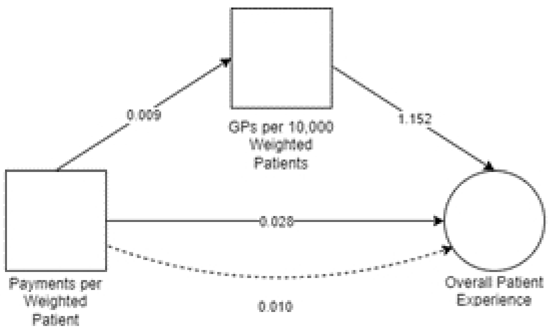

Figure 5

184x108mm (38 x 38 DPI)

The RECORD statement – checklist of items, extended from the STROBE statement, that should be reported in observational studies using routinely collected health data.

	Item No.	STROBE items	Location in manuscript where items are reported	RECORD items	Location in manuscript where items are reported
**Title and abstract**					
	1	(a) Indicate the study's design with a commonly used term in the title or the abstract (b) Provide in the abstract an informative and balanced summary of what was done and what was found		RECORD 1.1: The type of data used should be specified in the title or abstract. When possible, the name of the databases used should be included.<br><br>RECORD 1.2: If applicable, the geographic region and timeframe within which the study took place should be reported in the title or abstract.<br><br>RECORD 1.3: If linkage between databases was conducted for the study, this should be clearly stated in the title or abstract.	Methods<br><br>pg 4
**Introduction**					
Background rationale	2	Explain the scientific background and rationale for the investigation being reported			
Objectives	3	State specific objectives, including any prespecified hypotheses			
**Methods**					
Study Design	4	Present key elements of study design early in the paper			
Setting	5	Describe the setting, locations, and relevant dates, including periods of recruitment, exposure, follow-up, and data collection			

For peer review only

Participants	6	*(a) Cohort study* - Give the eligibility criteria, and the sources and methods of selection of participants. Describe methods of follow-up<br>*Case-control study* - Give the eligibility criteria, and the sources and methods of case ascertainment and control selection. Give the rationale for the choice of cases and controls<br>*Cross-sectional study* - Give the eligibility criteria, and the sources and methods of selection of participants<br><br>*(b) Cohort study* - For matched studies, give matching criteria and number of exposed and unexposed<br>*Case-control study* - For matched studies, give matching criteria and the number of controls per case		RECORD 6.1: The methods of study population selection (such as codes or algorithms used to identify subjects) should be listed in detail. If this is not possible, an explanation should be provided.<br><br>RECORD 6.2: Any validation studies of the codes or algorithms used to select the population should be referenced. If validation was conducted for this study and not published elsewhere, detailed methods and results should be provided.<br><br>RECORD 6.3: If the study involved linkage of databases, consider use of a flow diagram or other graphical display to demonstrate the data linkage process, including the number of individuals with linked data at each stage.	Methods<br><br>pg 4
Variables	7	Clearly define all outcomes, exposures, predictors, potential confounders, and effect modifiers. Give diagnostic criteria, if applicable.		RECORD 7.1: A complete list of codes and algorithms used to classify exposures, outcomes, confounders, and effect modifiers should be provided. If these cannot be reported, an explanation should be provided.	Methods<br><br>pg 4 and 5
Data sources/ measurement	8	For each variable of interest, give sources of data and details of methods of assessment (measurement).<br>Describe comparability of assessment methods if there is more than one group			

For peer review only

Bias	9	Describe any efforts to address potential sources of bias			
Study size	10	Explain how the study size was arrived at			
Quantitative variables	11	Explain how quantitative variables were handled in the analyses. If applicable, describe which groupings were chosen, and why			
Statistical methods	12	(a) Describe all statistical methods, including those used to control for confounding<br>(b) Describe any methods used to examine subgroups and interactions<br>(c) Explain how missing data were addressed<br>(d) *Cohort study* - If applicable, explain how loss to follow-up was addressed<br>*Case-control study* - If applicable, explain how matching of cases and controls was addressed<br>*Cross-sectional study* - If applicable, describe analytical methods taking account of sampling strategy<br>(e) Describe any sensitivity analyses			
Data access and cleaning methods		..		RECORD 12.1: Authors should describe the extent to which the investigators had access to the database population used to create the study population.	Methods<br>pg 4

For peer review only

				RECORD 12.2: Authors should provide information on the data cleaning methods used in the study.	
Linkage		..		RECORD 12.3: State whether the study included person-level, institutional-level, or other data linkage across two or more databases. The methods of linkage and methods of linkage quality evaluation should be provided.	Methods<br><br>pg 4
**Results**					
Participants	13	(a) Report the numbers of individuals at each stage of the study (*e.g.*, numbers potentially eligible, examined for eligibility, confirmed eligible, included in the study, completing follow-up, and analysed)<br>(b) Give reasons for non-participation at each stage.<br>(c) Consider use of a flow diagram		RECORD 13.1: Describe in detail the selection of the persons included in the study (*i.e.*, study population selection) including filtering based on data quality, data availability and linkage. The selection of included persons can be described in the text and/or by means of the study flow diagram.	Methods<br><br>Abstract pg 1
Descriptive data	14	(a) Give characteristics of study participants (*e.g.*, demographic, clinical, social) and information on exposures and potential confounders<br>(b) Indicate the number of participants with missing data for each variable of interest<br>(c) *Cohort study* - summarise follow-up time (*e.g.*, average and total amount)			
Outcome data	15	*Cohort study* - Report numbers of outcome events or summary measures over time<br>*Case-control study* - Report numbers in each exposure			

		category, or summary measures of exposure<br>*Cross-sectional study* - Report numbers of outcome events or summary measures			
Main results	16	(a) Give unadjusted estimates and, if applicable, confounder-adjusted estimates and their precision (e.g., 95% confidence interval). Make clear which confounders were adjusted for and why they were included<br>(b) Report category boundaries when continuous variables were categorized<br>(c) If relevant, consider translating estimates of relative risk into absolute risk for a meaningful time period			
Other analyses	17	Report other analyses done—e.g., analyses of subgroups and interactions, and sensitivity analyses			
**Discussion**					
Key results	18	Summarise key results with reference to study objectives			
Limitations	19	Discuss limitations of the study, taking into account sources of potential bias or imprecision. Discuss both direction and magnitude of any potential bias		RECORD 19.1: Discuss the implications of using data that were not created or collected to answer the specific research question(s). Include discussion of misclassification bias, unmeasured confounding, missing data, and changing eligibility over time, as they pertain to the study being reported.	Discussion<br>pg 10
Interpretation	20	Give a cautious overall interpretation of results considering objectives,			

For peer review only - http://bmjopen.bmj.com/site/about/guidelines.xhtml

		limitations, multiplicity of analyses, results from similar studies, and other relevant evidence			
Generalisability	21	Discuss the generalisability (external validity) of the study results			
**Other Information**					
Funding	22	Give the source of funding and the role of the funders for the present study and, if applicable, for the original study on which the present article is based			
Accessibility of protocol, raw data, and programming code		..		RECORD 22.1: Authors should provide information on how to access any supplemental information such as the study protocol, raw data, or programming code.	pg 13

*Reference: Benchimol EI, Smeeth L, Guttmann A, Harron K, Moher D, Petersen I, Sørensen HT, von Elm E, Langan SM, the RECORD Working Committee. The REporting of studies Conducted using Observational Routinely-collected health Data (RECORD) Statement. *PLoS Medicine* 2015; in press.

*Checklist is protected under Creative Commons Attribution (CC BY) license.

For peer review only

Does workforce explain the relationship between funding and patient experience? A mediation analysis of primary care data in England.

Journal:	*BMJ Open*
Manuscript ID	bmjopen-2023-072498.R1
Article Type:	Original research
Date Submitted by the Author:	27-Nov-2023
Complete List of Authors:	Salant, Natasha; University of Cambridge, Public Health and Primary Care<br>Massou , Efthalia ; University of Cambridge, Public Health and Primary Care<br>Awan, Hassan; Keele University<br>Ford, John; University of Cambridge, Public Health
**Primary Subject Heading**:	Public health
Secondary Subject Heading:	Health policy
Keywords:	STATISTICS & RESEARCH METHODS, Quality in health care < HEALTH SERVICES ADMINISTRATION & MANAGEMENT, Primary Health Care

I, the Submitting Author has the right to grant and does grant on behalf of all authors of the Work (as defined in the below author licence), an exclusive licence and/or a non-exclusive licence for contributions from authors who are: i) UK Crown employees; ii) where BMJ has agreed a CC-BY licence shall apply, and/or iii) in accordance with the terms applicable for US Federal Government officers or employees acting as part of their official duties; on a worldwide, perpetual, irrevocable, royalty-free basis to BMJ Publishing Group Ltd ("BMJ") its licensees and where the relevant Journal is co-owned by BMJ to the co-owners of the Journal, to publish the Work in this journal and any other BMJ products and to exploit all rights, as set out in our licence.

The Submitting Author accepts and understands that any supply made under these terms is made by BMJ to the Submitting Author unless you are acting as an employee on behalf of your employer or a postgraduate student of an affiliated institution which is paying any applicable article publishing charge ("APC") for Open Access articles. Where the Submitting Author wishes to make the Work available on an Open Access basis (and intends to pay the relevant APC), the terms of reuse of such Open Access shall be governed by a Creative Commons licence – details of these licences and which Creative Commons licence will apply to this Work are set out in our licence referred to above.

Other than as permitted in any relevant BMJ Author's Self Archiving Policies, I confirm this Work has not been accepted for publication elsewhere, is not being considered for publication elsewhere and does not duplicate material already published. I confirm all authors consent to publication of this Work and authorise the granting of this licence.

For peer review only

Does workforce explain the relationship between funding and patient experience? A mediation analysis of primary care data in England.

Natasha Salant[1]

Efthalia (Lina) Massou[1]

Hassan Awan[2]

John Ford[1]

[1] University of Cambridge

[2] University of Keele

Corresponding author: Natasha Salant, University of Cambridge MPhil Population Health Sciences 2021-2022, natashasalant@gmail.com

For peer review only

ABSTRACT

Objectives: To determine whether GP workforce contributes to the link between practice funding and patient experience. Specifically, to determine whether increased practice funding is associated with better patient experience, and to what degree an increase in workforce accounts for this relationship.

Setting: Primary care practice level analysis of workforce, funding and patient experience of all NHS practices in England.

Primary and secondary outcome measures: The link between NHS-provided funding to general practice (payments per patient) and patient experience, as per the General Practice Patient Survey, was evaluated. Subsequently, mediation analysis, adjusted for covariates, was used to scrutinise the extent to which GP workforce accounts for this relationship (measured as the number of GPs per 10,000 patients).

Participants: We included all general practices in England for which there was relevant data for each primary variable. Atypical practices were excluded, such as those with a patient list size of 0 or where the workforce variable was recorded as being more than 3 SD from the mean. After exclusion, 6139 practices were included in the final analysis.

Results: We found that workforce (GPs per 10,000 population) significantly ($p<0.001$) acts as a mediator in the effect of practice funding on overall patient experience even after adjusting for rurality, sex and age, and deprivation. On average, the mediated effect constitutes 30% of the total effect of practice funding on patient experience.

Conclusions: The increase in the number of doctors in primary care in England appears to be a mechanism through which augmented practice funding could positively impact patient experience. Policy initiatives targeting improved patient experience should prioritise considerations related to workforce and practice funding.

STRENGTHS AND LIMITATIONS

- This study is the first to explore the association between funding, workforce and patient experience through a mediation analysis.
- Given that publicly available datasets are used for this analysis, the data quality of the analysis suffers the same quality issues as these datasets. For instance, there is known inflation of full-time equivalent figures in the NHS Workforce dataset.
- The funding data is limited to NHS payments to practices and therefore does not capture other payments received by practices, for instance for training and teaching.

For peer review only

INTRODUCTION

In recent years, primary care services in England have been subject to increasing demand, tighter budgets, and workforce shortages (1-5). GP workforce has been found to be associated with better self-reported quality of care (6) and both GP and patient satisfaction (7). Workforce inequalities have been identified in previous research with the distribution of general practitioners (GPs), paramedics, and other allied health professionals favouring more affluent areas (7-10)

Patient experience is an important measure of primary health quality because it provides information about the process of care, for instance waiting times and interaction with staff, which cannot be replaced by other indicators (11). Self-reported continuity of care has been linked to cost-effectiveness, reduced emergency hospital admissions, and reduced mortality (12). Historically, patient satisfaction of general practice in the UK has been high; but it has been in decline since 2015 and the gap in patient satisfaction between socio-economic groups have been widening (1,2). On most measures of quality, practices in more deprived areas fare worse: they have lower quality scores, as measured by the Quality Outcomes Framework (QOF), and are more likely to be rated as 'inadequate' by the Care Quality Commission (1).

It has been suggested that funding inequalities are a key driving factor behind inequalities in workforce and satisfaction(1,13). Practices in more deprived areas receive 7% less general practice funding(1). Capitation funding for general practice is positively associated with overall practice quality and Care Quality Commission ratings (14). In addition, there is evidence of an association between funding and patient experience, whereby practices that receive less funding have lower levels of satisfaction(2). However, as it stands, there has been no robust investigation of the mechanisms of this relationship; in other words how lower funding leads to worse patient experience.

Mediation analysis is a statistical method that can be used to evaluate relationships between variables by quantifying the intermediary process through which a predictor variable affects an outcome variable. By using a mediation analysis, we can explore whether the relationship between funding and patient experience could be explained by GP supply. Put simply, we can evaluate whether practices which receive more funding employ more staff, and in turn whether they have better patient reported experience. The impact of socioeconomic deprivation and patient need on the relationships between these variables can also be explored.

For peer review only

METHODS

Data Sources

Four publicly available datasets were used: 'NHS Payments to General Practice' (15), 'General Practice Workforce' (16), GP Patient Survey Data (17), and 'National General Practice Profiles' (18).

Funding data consisted of payments from the NHS to individual general practices in England as part of their contract. Total NHS payments made to general practices included the core capitation amount (global sum) and other payment categories including financial incentives (QOF) and payments for premises. The dataset included total payment and payment per registered and weighted patients (calculated using the Carr-Hill formula (19)).

General Practice workforce data are uploaded by practices every quarter and the database covers all general practices in England. GP data are available for Full Time Equivalent (FTE) across all GP partners, salaried GPs, training grades and locums.

The GP Patient Survey (GPPS) is a national self-reported patient experience survey of over 900,000 adults per year in England (20). It is commissioned by NHS England with the aim of monitoring quality of care. The results are weighted by age, sex, region and socioeconomic status to ensure representativeness (2). Survey results provided practice-level data that is comparable across practices and time (21).

National General Practice Profiles are a set of approximately 150 general practice level indicators that are produced by the Office for Health Improvement and Disparities for practices across England. The Index of Multiple Deprivation (IMD) score is an area-based measure of socio-economic deprivation that comprises of seven distinct weighted domains: Income (22.5%), Employment (22.5%), Health Deprivation and Disability (13.5%), Education, Skills and Training (13.5%), Crime (9.3%), Barriers to Housing and Services (9.3%), and Living Environment (9.3%) (22).

Variables

Predictor

For funding we used payment per registered patient or per weighted patient by dividing 'total NHS payment' by 'registered patient' or 'weighted patient'.

Outcome

We used four patient experience variables from the GP Patient Survey based on previous studies(2,13).

(a) proportion of respondents who had a good or very good experience of making an appointment (Access),
(b) proportion of respondents who always see their preferred GP (Continuity),
(c) proportion of respondents who had trust and confidence in their GP (Trust), and
(d) proportion of respondents who had an overall good or very good experience (Overall Experience).

Mediator

We used full time equivalent (FTE) GPs per 10,000 registered or weighted patients as the mediator.

For peer review only

Covariates

An adjusted model was undertaken using patient weights and covariates. Weighted payments per patient takes into account patient sex, age, long-standing health condition and rurality, so these were not added as separate variables. IMD was also added as a covariate.

Sample

As illustrated in Figure 1Sup, we included all general practices in England with data available. Where practices were entirely missing from payments, workforce and patient experience datasets, they were excluded. In addition, atypical practices were excluded, for example if the patient list size was 0 or where workforce data were improbable (defined as more the 3 standard deviations from the mean).

Data merge

Relevant variables from data sources were linked using practice code, and sense-checked by scanning that practice names matched. To account for the lag between funding, employment and patient experience, 2017/18 funding data were used, December 2018 workforce data and March 2019 patient experience data. Pandemic data were not used because of data quality issues and challenges around accounting for the impact of changes to services.

Statistical analysis

Three mediation model structures were built using the Baron and Kenny approach (23) also known as the causal steps approach, and all models assumed the causal structure in Figures 1 and 2. These were: an unadjusted model (where GPs per registered patient was tested as a mediator of the effect of payments per registered patient on patient experience), a weighted patients' model (where GPs per weighted patient was tested as a mediator of payments per weighted patient on patient experience) and a deprivation adjusted model (where the unadjusted model was adjusted for deprivation). The weighted model was weighted using the Carr-Hill formula which takes into account, patient sex, age, long-standing health condition and rurality. For each model structure, there were four distinct models that tested each distinct patient experience variable.

In total, twelve mediation models were tested. For each, a simple linear regression of the predictor on the outcome was run to test whether there was a significant association. Mediation analysis was considered if the outcome variable was significantly associated with the predictor variable, the mediator variable was significantly associated with the predictor variable, and controlling for the predictor variable, that the mediator variable associated with the outcome variable. The significance of indirect effect was tested using bootstrapping procedures. Unstandardised indirect effects were computed for each of 1000 bootstrapped samples, and the 95% confidence interval was computed by determining the indirect effects at the 2.5th and 97.5th percentiles. If mediation was established using simple linear regressions, the model was adjusted for patient sex, age, long-standing health condition and rurality (24,25).

In addition, the model's sensitivity to removing outliers was tested by running the same analyses on datasets that did not remove outliers.

All analyses were conducted using R (version 4.2) and RStudio.

For peer review only

Patient and Public Involvement

No patient involved.

For peer review only

RESULTS

The final number of practices included ranged from 5,946 to 6,139 (Table 1). The continuity dataset included the fewest practices because the survey's continuity question was answered by 2% fewer participants than access, trust and overall experience questions. Across all datasets, the average practice receives NHS payments of £152 per registered patient and the average practice size is 8,640 patients. Most practices have a General Medical Services (GMS) contract, do not dispense and are based in urban areas (Table 1Sup presents extended practice characteristics). Practices employ on average 5.6 FTE GPs. The average practice receives a good or very good rating of 83% for overall patient experience, 69% for access, 49% for continuity and 95% for trust.

Table 1: Practice characteristics for each outcome dataset

	Access	Continuity	Trust	Overall Experience
	Total (N=6137)	Total (N=5946)	Total (N=6137)	Total (N=6139)
Payments per Patient (£) Mean (SD)	152 (40.3)	152 (39.4)	152 (40.3)	152 (40.3)
Payments per Weighted Patient (£) Mean (SD)	151 (35.4)	151 (34.7)	151 (35.4)	151 (35.4)
Rurality n (%)				
Rural	919 (15.0%)	885 (14.9%)	919 (15.0%)	919 (15.0%)
Urban	5218 (85.0%)	5061 (85.1%)	5218 (85.0%)	5220 (85.0%)
Registered Patients Mean (SD)	8640 (5220)	8790 (5150)	8640 (5220)	8640 (5220)
FTE GPs per 10,000 patients (pts) Mean (SD)	5.61 (2.20)	5.62 (2.19)	5.61 (2.20)	5.61 (2.20)
Total Patient number > 65 yrs Mean (SD)	1620 (1210)	1650 (1200)	1620 (1210)	1620 (1210)
% Patients with long-standing health condition Mean (SD)	51.3 (8.58)	51.3 (8.58)	51.3 (8.58)	51.3 (8.59)
Patient Experience rated good (%) Mean (SD)	69.1 (14.4)	49.1 (18.5)	95.3 (3.80)	83.4 (9.74)
[Min, Max]	[19.1, 100]	[2.14, 98.0]	[71.6, 100]	[32.2, 100]
Response Rate (%) Mean (SD)	35.9 (10.8)	36.0 (10.8)	35.9 (10.8)	35.8 (10.8)

<u>*Does workforce mediate the relationship between funding and overall patient outcomes?*</u>

Unadjusted analysis

In the unadjusted model, the effect of funding on overall patient experience was partially mediated via GPs per 10,000 patients (Figure 3). The proportion of the relationship between funding and overall patient experience that is mediated by GPs per 10,000 is 32%. In other words, the increase in payments per patient that drives an increase in GPs per 10,000 patients explains more than one third of the effect on an increase in overall patient experience.

The regression coefficient between funding and overall patient experience was statistically significant, as was the regression coefficient between GPs per 10,000 patients and overall patient experience (p<0.001). The total effect of funding on overall patient experience was 0.044. The indirect effect via workforce was 0.014 (95%CI 0.011 to 0.017). The model could be seen to illustrate that a £10 increase in practice funding is associated with a 0.44% increase in overall patient experience.

Analysis adjusted for socio-economic deprivation

When adjusted for socio-economic deprivation, the effect of funding on overall patient experience is still significantly (p<0.001) partially mediated by GPs per 10,000 patients (Figure 4). In this adjusted model, the indirect effect via GP workforce was 0.013 (95% CI, 0.011 to 0.015). The total effect of funding on overall patient experience (0.043) has the same effect size as the unadjusted model and is still significant (p<0.001). However, the direct effect (0.030) and indirect effect (0.013), while still both significant (p<0.001), differ slightly in size from the unadjusted model, and the proportion mediated is 30%.

Analysis using weighted funding data

Figure 5 shows the effect of funding on overall patient experience was partially mediated via GPs per 10,000 weighted patients. The indirect effect via workforce was 0.010 (95%CI 0.008 to 0.012). This weighted model could be seen to illustrate that a £10 increase in practice funding is associated with a 0.38% increase in overall patient experience, and the proportion of the total effect that weighted funding has on overall patient experience is 26%.

The relationship between weighted funding and overall patient experience (0.028) was significant and similar to the unadjusted model, as was the relationship between GPs per 10,000 patients and overall patient experience (1.152).

<u>*Does workforce mediate the relationship between funding and access, trust and continuity?*</u>

Unadjusted analysis

Mediation models with access, trust and continuity indicators as outcome variables were all similarly significantly mediated (p<0.001) by GPs per 10,000 patients (Table 2Sup). The 'access' outcome had the strongest association between funding and patient experience, with the unadjusted model demonstrating that a £10 pound increase in practice funding was associated with a 0.44% increase in patient experience, of which 36% could be explained by an increase of 1 GP per 10,000 patients.

Adjusted analysis

When using weighted data and adjusting for deprivation, the 'access' effect size remained the highest out of all models, with GP workforce accounting for ~31% of the effect size. Direct, total and mediated effect sizes were substantially smaller for the continuity and trust models indicating that

For peer review only

while an increase in GPs per 10,000 patients was a statistically significant mediator for trust and continuity measures of patient experience, the effect may be more relevant for access and overall experience measures.

Sensitivity analyses including the data outliers did not have a substantial impact on the results.

For peer review only

Main finding of this study

We found that the number of GPs per 10,000 patients significantly ($p<0.001$) mediated the effect of practice funding on overall patient experience, access, trust and continuity. In simpler terms, practices with more funding had better patient experience and some of this relationship can be accounted for by an increase in workforce. However, it is crucial to note that the effect sizes are relatively modest. In the unadjusted model, the total effect size of the association between payments per patient and overall patient experience is 0.044. This implies that a £10 increase in payments per patient is linked to a 0.4% rise in overall patient experience. Considering the mean payment per patient is £152, an average practice would experience only a marginal increase in overall patient experience, even with a funding boost of 6%. Nonetheless, our findings indicate that 30% of the explanation for the link between an increase in payments per patient and improved overall patient experience is attributed to an increase in FTE GPs per 10,000 patients.

Strengths and limitations

The study was strengthened by building up the mediation model step by step, allowing an understanding of the associations at each level of the mediation. In addition, we used a non-parametric estimation of the indirect effect and significance, which, because of its wide applicability to a variety of models, resolves any doubts arising from whether or not the method of estimation and significance testing is suitable for this model (24, 25).

GP Patient Survey response rate is about 30%, raising questions about whether the data is suitable for a quantitative England-wide study. However, because responses are weighted to account for selection bias, demographic characteristics of the eligible population, as well as differences between responders and non-responders (26), this problem is mitigated. In addition, we have only identified four patient experience outcomes for this study, meaning there may be other components which had different results. For example, waiting times may have a stronger relationship with workforce and funding.

Both the General Practice Workforce and NHS Payments datasets have known quality considerations. For the Workforce dataset, there is known inflation of FTE and headcount figures due to the recruitment of temporary staff to cover staff on long-term leave (27). For the NHS Payments dataset, patient list numbers should be viewed with caution given that there is known list inflation whereby patients who have left a practice are not deregistered (28).

This analysis of practice funding is limited to an analysis of NHS payments to practice and does not include practice funding received from other sources such as research or training. However, this limitation addresses a wider problem that practice-level data on additional funding, expenditure and profit data is not publicly available.

What this study adds

This study is the first to explore the association between practice-level NHS payments per patient, patient experience and workforce through mediation analyses. Despite prior evidence to show that an increase in NHS practice funding is associated with an increase in patient experience (2) and CQC scores (12), it is unclear from these studies how increasing practice funding may lead to improved quality outcomes. Understanding the mechanisms of action is valuable to recognise correlates of increasing GP per patient ratios, identify practices that may be more or less likely to benefit from an

For peer review only

increase in payments per patient, or inform policy so that increasing practice funding can be more strategic. Payments per weighted patient are not equal per decile, with more affluent deciles receiving more funding per weighted patient. That information is enriched by the finding that the inequality in payments is associated with an inequality in capability to recruit GPs, which plays a substantial role in patient experience.

Moreover, this study read together with previous analyses on the widening inequality GP distribution (9, 10) should be interpreted to mean that if the status quo continues with regard to NHS payments to practices, i.e. a total increase in average payments per patient of approximately 4% over the past four years (29), the inequality in payments per weighted patient, as well as that in GPs per 10,000 weighted patients, will likely continue to grow. Given the results of this study, it is unsurprising that recent results from the GP Patient Survey reveal a widening gap in patient experience, with patients attending practices in more deprived neighbourhoods reporting worse overall experience than patients attending practices in more affluent neighbourhoods (30).

Investment in general practice in England is thought to have lagged behind what is necessary to finance increasing total expenditure, population growth, increasing consultations and expanding complexity of health needs (29). While undeniable value for money can be achieved with primary care investment (31, 32), the results of this study do not suggest that simply investing more money in practices would solve the problem. While practice payments per patient significantly impact the number of GPs per 10,000 patients, the effect size is small. This reflects that workforce recruitment is a problem that is not entirely dependent on practice funding. However, if the system is committed to improving quality, policy must target both practice funding, GP recruitment and quality improvement.

Policy recommendations

Review of the contractual funding model (Carr-Hill formula) should be reviewed to address the unequal distribution of payments per weighted patient. Its inaccurate reflection of patient need is identified in the inequality of average practice payments per weighted patients, and according to this analysis this has an impact on the distribution of GPs per 10,000 patients as well as on inequality of patient experience. In addition, given the effect of practice funding on GPs per 10,000 patients identified by this study, as well as the widening inequality of GP distribution found in multiple previous studies, it is recommended that additional funding is identified and targeted towards underdoctored areas to stop the inequality from increasing. This targeted investment could have a knock-on effect on patient experience in these areas, according to the findings of this study. Finally, given the demonstrable link between funding, GP supply and patient experience, it is recommended that practices achieving low quality of care scores are not punished through missing out on financial incentives; but rather practices are supported to improve quality of care.

CONCLUSION

We found that the number of GPs per 10,000 patients is a significant mediator and explains a third of the relationship between funding and patient experience. However the effect is small, meaning that a substantial increase in funding would be required to meaningfully improve patient experience. Funding increases should therefore be implemented alongside policy reform to improve patient experience and reduce inequalities.

For peer review only

Figure Legend

Figure 1: Assumed causal structure of unadjusted mediation model

Figure 2: Assumed causal structure of adjusted mediation model

Figure 3: Unadjusted mediation model results

Figure 4: Deprivation adjusted mediation model results

Figure 5: Carr-Hill adjusted mediation model results

Supplemental Material

Figure 1Sup: Data sample flow chart

Table 1Sup: Extended practice characteristics for each outcome dataset

Table 2Sup: Access, Continuity and Trust mediation models

Contributorship statement

Natasha Salant is the primary author having made substantial contributions to the conception and design of the work, the acquisition of the data, the cleaning and analysis of the data as well as the interpretation thereof. She is also responsible for drafting of the work, revising it, and final approval for it to be published, thereby agreeing to be accountable for all aspects of the work. Hassan Awan made substantial contributions to the interpretation of data, the critical revision and final approval for publication thereby agreeing to be accountable for the work. Efthalia Massou made substantial contributions to the design of the statistical analysis as well as the interpretation of the analysis, critical revision of the draft, and final approval for publication thereby being accountable for the work. John Ford conceptualised the initial idea and made substantial contributions to the concept and design of the work as well as to the interpretation of the data, critical revision of the draft, and final approval for publication thereby agreeing to be accountable for the work.

Competing Interests

No authors have any conflicting interests to declare.

For peer review only

Funding

Natasha Salant is grateful to Chevening Fellowships, the UK government's global scholarship programme, funded by the Foreign, Commonwealth and Development Office (FCDO) and partner organisations for the funding of her MPhil program, thereby making this research possible.

Data sharing statement

Datasets used are available publicly as per references provided for each dataset used. Code for analysis is available upon request.

Ethics Approval

This study does not involve human participants and ethical approval was not required.

For peer review only

References

1. Level or not? - The Health Foundation [Internet]. [cited 2022 Jan 1]. Available from: https://www.health.org.uk/publications/reports/level-or-not

2. L'Esperance V, Gravelle H, Schofield P, Ashworth M. Impact of primary care funding on patient satisfaction: a retrospective longitudinal study of English general practice, 2013–2016. Br J Gen Pract. 2020 Dec 1;71(702):e47–54.

3. The bigger picture - The Health Foundation [Internet]. [cited 2022 Jul 16]. Available from: https://www.health.org.uk/publications/reports/the-bigger-picture

4. Tackling the inverse care law - The Health Foundation [Internet]. [cited 2022 Jul 8]. Available from: https://www.health.org.uk/publications/reports/tackling-the-inverse-care-law

5. 'Levelling up' general practice in England - The Health Foundation [Internet]. [cited 2022 Jun 24]. Available from: https://www.health.org.uk/publications/long-reads/levelling-up-general-practice-in-england

6. Vallejo-Torres L, Morris S. Primary care supply and quality of care in England. Eur J Health Econ. 2018;19(4):499–519.

7. Gibson J, Francetic I, Spooner S, Checkland K, Sutton M. Primary care workforce composition and population, professional, and system outcomes: a retrospective cross-sectional analysis. Br J Gen Pract. 2022 May 1;72(718):e307–15.

8. Cookson R, Propper C, Asaria M, Raine R. Socio-Economic Inequalities in Health Care in England. Fisc Stud. 2016;37(3–4):371–403.

9. Nussbaum C, Massou E, Fisher R, Morciano M, Harmer R, Ford J. Inequalities in the distribution of the general practice workforce in England: a practice-level longitudinal analysis. BJGP Open [Internet]. 2021 Oct 1 [cited 2022 Apr 12];5(5). Available from: https://bjgpopen.org/content/5/5/BJGPO.2021.0066

10. Asaria M, Cookson R, Fleetcroft R, Ali S. Unequal socioeconomic distribution of the primary care workforce: whole-population small area longitudinal study. BMJ Open. 2016 Jan 19;6(1):e008783.

11. Schoenfelder T. Patient Satisfaction: A Valid Indicator for the Quality of Primary Care? Prim Health Care Open Access [Internet]. 2012 [cited 2022 Mar 23];02(04). Available from: https://www.omicsonline.org/open-access/patient-satisfaction-a-valid-indicator-for-the-quality-of-primary-care-2167-1079.1000e106.php?aid=8310

12. Pereira Gray DJ, Sidaway-Lee K, White E, Thorne A, Evans PH. Continuity of care with doctors—a matter of life and death? A systematic review of continuity of care and mortality. BMJ Open. 2018 Jun;8(6):e021161.

13. Saunders CL, Flynn S, Massou E, Lyratzopoulos G, Abel G, Burt J. Sociodemographic inequalities in patients' experiences of primary care: an analysis of the General Practice Patient Survey in England between 2011 and 2017. J Health Serv Res Policy. 2021 Jul 1;26(3):198–207.

For peer review only - http://bmjopen.bmj.com/site/about/guidelines.xhtml

14. L'Esperance V, Gravelle H, Schofield P, Santos R, Ashworth M. Relationship between general practice capitation funding and the quality of primary care in England: a cross-sectional, 3-year study. BMJ Open. 2019 Nov 7;9(11):e030624.

15. NHS Payments to General Practice - NHS Digital [Internet]. [cited 2022 Jan 31]. Available from: https://digital.nhs.uk/data-and-information/publications/statistical/nhs-payments-to-general-practice

16. General Practice Workforce [Internet]. NHS Digital. [cited 2022 Jan 31]. Available from: https://digital.nhs.uk/data-and-information/publications/statistical/general-and-personal-medical-services

17. Survey and Reports [Internet]. [cited 2022 Feb 3]. Available from: https://www.gp-patient.co.uk/surveysandreports

18. National General Practice Profiles - OHID [Internet]. [cited 2022 Feb 5]. Available from: https://fingertips.phe.org.uk/profile/general-practice

19. Dyson M. Global sum allocation formula [Internet]. The British Medical Association is the trade union and professional body for doctors in the UK. [cited 2021 Dec 8]. Available from: https://www.bma.org.uk/advice-and-support/gp-practices/funding-and-contracts/global-sum-allocation-formula

20. GP Patient Survey – About [Internet]. [cited 2022 Feb 3]. Available from: https://www.gp-patient.co.uk/About

21. Tallett A, Poots AJ, Graham C, Peters M, Corbett R, Sizmur S, et al. How can patient experience scores be used to predict quality inspection ratings? A retrospective cross-sectional study of national primary care datasets in the UK. BMJ Open. 2020 Nov 26;10(11):e041709.

22. English indices of deprivation [Internet]. GOV.UK. [cited 2022 Feb 9]. Available from: https://www.gov.uk/government/collections/english-indices-of-deprivation

23. Baron RM, Kenny DA. The moderator–mediator variable distinction in social psychological research: Conceptual, strategic, and statistical considerations. J Pers Soc Psychol. 1986 Dec;51(6):1173–82.

24. Imai K, Keele L, Yamamoto T. Identification, Inference and Sensitivity Analysis for Causal Mediation Effects. Stat Sci [Internet]. 2010 Feb 1 [cited 2022 Jul 13];25(1). Available from: https://projecteuclid.org/journals/statistical-science/volume-25/issue-1/Identification-Inference-and-Sensitivity-Analysis-for-Causal-Mediation-Effects/10.1214/10-STS321.full

25. Imai K, Keele L, Tingley D. A general approach to causal mediation analysis. Psychol Methods. 2010 Dec;15(4):309–34.

26. GPPS Technical Annex 2019 [Internet]. [cited 2022 Jul 14]. Available from: https://www.gp-patient.co.uk/downloads/2019/GPPS_2019_Technical_Annex_PUBLIC.pdf

27. Background Data Quality Statement [Internet]. NHS Digital. [cited 2022 Jul 15]. Available from: https://digital.nhs.uk/data-and-information/publications/statistical/general-and-personal-medical-services/31-october-2021/background-data-quality-statement

For peer review only

28. Executive Summary [Internet]. NHS Digital. [cited 2022 Jul 15]. Available from: https://digital.nhs.uk/data-and-information/publications/statistical/nhs-payments-to-general-practice/england-2020-21/executive-summary-copy

29. Dyson M. Investment in general practice [Internet]. The British Medical Association is the trade union and professional body for doctors in the UK. [cited 2022 Jul 27]. Available from: https://www.bma.org.uk/advice-and-support/nhs-delivery-and-workforce/funding/investment-in-general-practice

30. Statistics » GP Patient Survey 2022 [Internet]. [cited 2022 Jul 15]. Available from: https://www.england.nhs.uk/statistics/2022/07/14/gp-patient-survey-2022/#

31. Starfield B, Shi L, Macinko J. Contribution of Primary Care to Health Systems and Health. Milbank Q. 2005;83(3):457–502.

32. Schäfer WLA, Boerma WGW, van den Berg MJ, De Maeseneer J, De Rosis S, Detollenaere J, et al. Are people's health care needs better met when primary care is strong? A synthesis of the results of the QUALICOPC study in 34 countries. Prim Health Care Res Dev. 2019 Jul 1;20:e104.

For peer review only

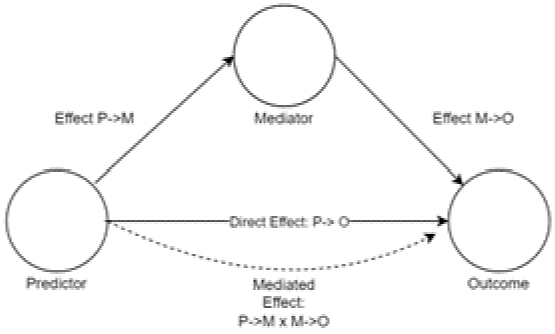

Figure 1

191x118mm (38 x 38 DPI)

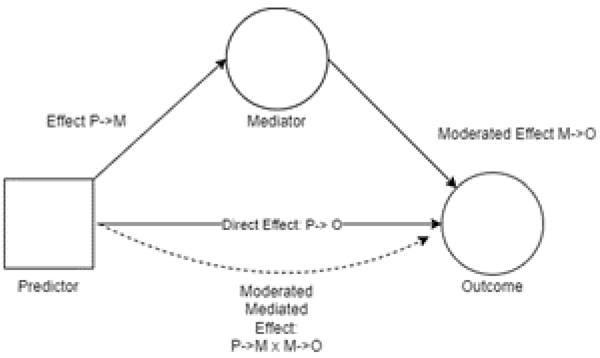

Figure 2

207x118mm (38 x 38 DPI)

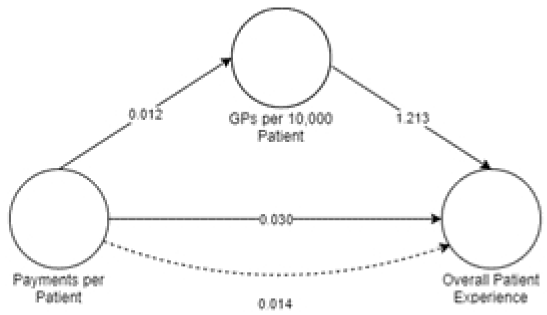

Figure 3

181x101mm (38 x 38 DPI)

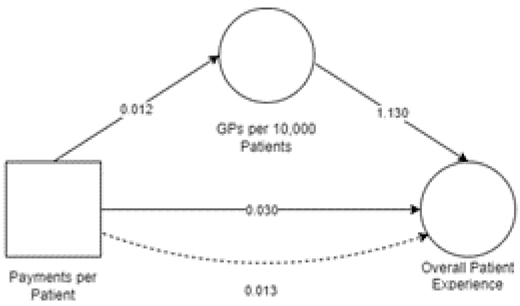

Figure 4

174x102mm (38 x 38 DPI)

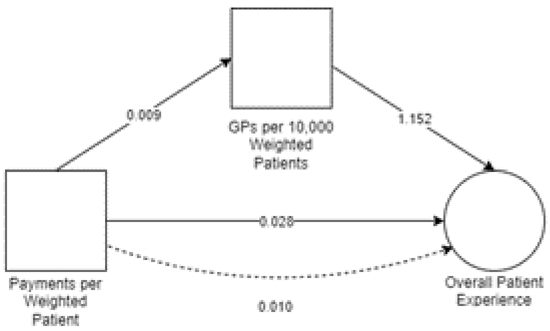

Figure 5

184x108mm (38 x 38 DPI)

Figure 1Sup: Data sample flow chart

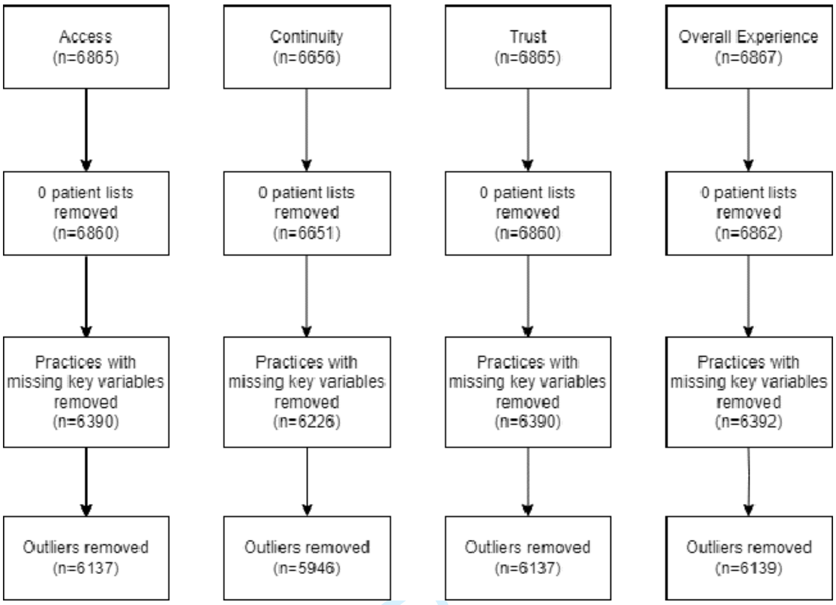

For peer review only

Table 1Sup: Extended practice characteristics for each outcome dataset

	Access	*Continuity*	*Trust*	*Overall Experience*
	Total (N=6137)	Total (N=5946)	Total (N=6137)	Total (N=6139)
Total NHS Payments (£) *Mean (SD)*	1,300,000 (806,000)	1,320,000 (794,000)	1,300,000 (806,000)	1,300,000 (806,000)
Payments per Patient (£) *Mean (SD)*	152 (40.3)	152 (39.4)	152 (40.3)	152 (40.3)
Payments per Weighted Patient (£) *Mean (SD)*	151 (35.4)	151 (34.7)	151 (35.4)	151 (35.4)
Contract Type n (%)				
APMS	136 (2.2%)	125 (2.1%)	136 (2.2%)	136 (2.2%)

GMS	4313 (70.3%)	4176 (70.2%)	4313 (70.3%)	4315 (70.3%)
PMS	1688 (27.5%)	1645 (27.7%)	1688 (27.5%)	1688 (27.5%)
Unknown	0 (0%)	0 (0%)	0 (0%)	0 (0%)
Dispensing n (%)				
Yes	860 (14.0%)	833 (14.0%)	860 (14.0%)	860 (14.0%)
No	5277 (86.0%)	5113 (86.0%)	5277 (86.0%)	5279 (86.0%)
Unknown	0 (0%)	0 (0%)	0 (0%)	0 (0%)
Rurality n (%)				
Rural	919 (15.0%)	885 (14.9%)	919 (15.0%)	919 (15.0%)
Urban	5218 (85.0%)	5061 (85.1%)	5218 (85.0%)	5220 (85.0%)
Registered Patients Mean (SD)	8640 (5220)	8790 (5150)	8640 (5220)	8640 (5220)
Weighted Patients Mean (SD)	8630 (5120)	8780 (5060)	8630 (5120)	8630 (5120)
FTE GPs per 10,000 patients (pts) Mean (SD)	5.61 (2.20)	5.62 (2.19)	5.61 (2.20)	5.61 (2.20)
Total Patient number > 65 yrs Mean (SD)	1620 (1210)	1650 (1200)	1620 (1210)	1620 (1210)
% Patients with long-standing health condition Mean (SD)	51.3 (8.58)	51.3 (8.58)	51.3 (8.58)	51.3 (8.59)

For peer review only

Patient Experience rating (%)<br>*Mean (SD)*<br>*[Min, Max]*	69.1 (14.4)<br>[19.1, 100]	49.1 (18.5)<br>[2.14, 98.0]	95.3 (3.80)<br>[71.6, 100]	83.4 (9.74)<br>[32.2, 100]
Response Rate (%)<br>*Mean (SD)*	35.9 (10.8)	36.0 (10.8)	35.9 (10.8)	35.8 (10.8)

Table 2Sup: Access, Continuity and Trust mediation models

	Access		Continuity		Trust	
	Coefficient	CI: 95%	Coefficient	CI: 95%	Coefficient	CI: 95%
Unadjusted Model						
**Direct Effect of predictor on outcome**	0.044	(0.037; 0.06)	0.044	(0.030; 0.06)	0.007	(0.006; 0.01)
**Total Effect of predictor on outcome**	0.062	(0.053; 0.07)	0.050	(0.036; 0.06)	0.013	(0.010; 0.01)
**Indirect (Mediated) Effect**	0.016	(0.013; 0.02)	0.006	(0.004; 0.01)	0.005	(0.004; 0.01)
Carr-Hill adjusted Model						
**Direct Effect of predictor on outcome**	0.048	(0.037; 0.06)	0.037	(0.022; 0.06)	0.007	(0.004; 0.01)

For peer review only

**Total Effect of predictor on outcome**	0.060	(0.048; 0.07)	0.041	(0.026; 0.06)	0.010	(0.0073; 0.01)
**Indirect (Mediated) Effect**	0.011	(0.009; 0.01)	0.004	(0.002; 0.01)	0.003	(0.0025; 0.0035)
Deprivation adjusted Model<br>**Direct Effect of predictor on outcome**	0.047	(0.038; 0.06)	0.045	(0.030; 0.06)	0.008	(0.006; 0.010)
**Total Effect of predictor on outcome**	0.061	(0.053; 0.07)	0.050	(0.035; 0.06)	0.012	(0.010; 0.014)
**Indirect (Mediated) Effect**	0.015	(0.012; 0.02)	0.005	(0.003; 0.01)	0.004	(0.003; 0.005)

For peer review only

The RECORD statement – checklist of items, extended from the STROBE statement, that should be reported in observational studies using routinely collected health data.

	Item No.	STROBE items	Location in manuscript where items are reported	RECORD items	Location in manuscript where items are reported
**Title and abstract**					
	1	(a) Indicate the study's design with a commonly used term in the title or the abstract (b) Provide in the abstract an informative and balanced summary of what was done and what was found		RECORD 1.1: The type of data used should be specified in the title or abstract. When possible, the name of the databases used should be included.<br><br>RECORD 1.2: If applicable, the geographic region and timeframe within which the study took place should be reported in the title or abstract.<br><br>RECORD 1.3: If linkage between databases was conducted for the study, this should be clearly stated in the title or abstract.	Methods<br><br>pg 4
**Introduction**					
Background rationale	2	Explain the scientific background and rationale for the investigation being reported			
Objectives	3	State specific objectives, including any prespecified hypotheses			
**Methods**					
Study Design	4	Present key elements of study design early in the paper			
Setting	5	Describe the setting, locations, and relevant dates, including periods of recruitment, exposure, follow-up, and data collection			

For peer review only

Participants	6	*(a) Cohort study* - Give the eligibility criteria, and the sources and methods of selection of participants. Describe methods of follow-up<br>*Case-control study* - Give the eligibility criteria, and the sources and methods of case ascertainment and control selection. Give the rationale for the choice of cases and controls<br>*Cross-sectional study* - Give the eligibility criteria, and the sources and methods of selection of participants<br><br>*(b) Cohort study* - For matched studies, give matching criteria and number of exposed and unexposed<br>*Case-control study* - For matched studies, give matching criteria and the number of controls per case		RECORD 6.1: The methods of study population selection (such as codes or algorithms used to identify subjects) should be listed in detail. If this is not possible, an explanation should be provided.<br><br>RECORD 6.2: Any validation studies of the codes or algorithms used to select the population should be referenced. If validation was conducted for this study and not published elsewhere, detailed methods and results should be provided.<br><br>RECORD 6.3: If the study involved linkage of databases, consider use of a flow diagram or other graphical display to demonstrate the data linkage process, including the number of individuals with linked data at each stage.	Methods<br><br>pg 4
Variables	7	Clearly define all outcomes, exposures, predictors, potential confounders, and effect modifiers. Give diagnostic criteria, if applicable.		RECORD 7.1: A complete list of codes and algorithms used to classify exposures, outcomes, confounders, and effect modifiers should be provided. If these cannot be reported, an explanation should be provided.	Methods<br><br>pg 4 and 5
Data sources/ measurement	8	For each variable of interest, give sources of data and details of methods of assessment (measurement).<br>Describe comparability of assessment methods if there is more than one group			

For peer review only

Bias	9	Describe any efforts to address potential sources of bias			
Study size	10	Explain how the study size was arrived at			
Quantitative variables	11	Explain how quantitative variables were handled in the analyses. If applicable, describe which groupings were chosen, and why			
Statistical methods	12	(a) Describe all statistical methods, including those used to control for confounding<br>(b) Describe any methods used to examine subgroups and interactions<br>(c) Explain how missing data were addressed<br>(d) *Cohort study* - If applicable, explain how loss to follow-up was addressed<br>*Case-control study* - If applicable, explain how matching of cases and controls was addressed<br>*Cross-sectional study* - If applicable, describe analytical methods taking account of sampling strategy<br>(e) Describe any sensitivity analyses			
Data access and cleaning methods		..		RECORD 12.1: Authors should describe the extent to which the investigators had access to the database population used to create the study population.	Methods<br>pg 4

For peer review only

				RECORD 12.2: Authors should provide information on the data cleaning methods used in the study.	
Linkage		..		RECORD 12.3: State whether the study included person-level, institutional-level, or other data linkage across two or more databases. The methods of linkage and methods of linkage quality evaluation should be provided.	Methods<br>pg 4
**Results**					
Participants	13	(a) Report the numbers of individuals at each stage of the study (*e.g.*, numbers potentially eligible, examined for eligibility, confirmed eligible, included in the study, completing follow-up, and analysed)<br>(b) Give reasons for non-participation at each stage.<br>(c) Consider use of a flow diagram		RECORD 13.1: Describe in detail the selection of the persons included in the study (*i.e.*, study population selection) including filtering based on data quality, data availability and linkage. The selection of included persons can be described in the text and/or by means of the study flow diagram.	Methods<br>Abstract pg 1
Descriptive data	14	(a) Give characteristics of study participants (*e.g.*, demographic, clinical, social) and information on exposures and potential confounders<br>(b) Indicate the number of participants with missing data for each variable of interest<br>(c) *Cohort study* - summarise follow-up time (*e.g.*, average and total amount)			
Outcome data	15	*Cohort study* - Report numbers of outcome events or summary measures over time<br>*Case-control study* - Report numbers in each exposure			

For peer review only

		category, or summary measures of exposure<br>*Cross-sectional study* - Report numbers of outcome events or summary measures			
Main results	16	(a) Give unadjusted estimates and, if applicable, confounder-adjusted estimates and their precision (e.g., 95% confidence interval). Make clear which confounders were adjusted for and why they were included<br>(b) Report category boundaries when continuous variables were categorized<br>(c) If relevant, consider translating estimates of relative risk into absolute risk for a meaningful time period			
Other analyses	17	Report other analyses done—e.g., analyses of subgroups and interactions, and sensitivity analyses			
**Discussion**					
Key results	18	Summarise key results with reference to study objectives			
Limitations	19	Discuss limitations of the study, taking into account sources of potential bias or imprecision. Discuss both direction and magnitude of any potential bias		RECORD 19.1: Discuss the implications of using data that were not created or collected to answer the specific research question(s). Include discussion of misclassification bias, unmeasured confounding, missing data, and changing eligibility over time, as they pertain to the study being reported.	Discussion<br>pg 10
Interpretation	20	Give a cautious overall interpretation of results considering objectives,			

For peer review only

		limitations, multiplicity of analyses, results from similar studies, and other relevant evidence			
Generalisability	21	Discuss the generalisability (external validity) of the study results			
**Other Information**					
Funding	22	Give the source of funding and the role of the funders for the present study and, if applicable, for the original study on which the present article is based			
Accessibility of protocol, raw data, and programming code		..		RECORD 22.1: Authors should provide information on how to access any supplemental information such as the study protocol, raw data, or programming code.	pg 13

*Reference: Benchimol EI, Smeeth L, Guttmann A, Harron K, Moher D, Petersen I, Sørensen HT, von Elm E, Langan SM, the RECORD Working Committee. The REporting of studies Conducted using Observational Routinely-collected health Data (RECORD) Statement. *PLoS Medicine* 2015; in press.

*Checklist is protected under Creative Commons Attribution (CC BY) license.

For peer review only

BMJ Open

Does workforce explain the relationship between funding and patient experience? A mediation analysis of primary care data in England.

Journal:	*BMJ Open*
Manuscript ID	bmjopen-2023-072498.R2
Article Type:	Original research
Date Submitted by the Author:	24-Jan-2024
Complete List of Authors:	Salant, Natasha; University of Cambridge, Public Health and Primary Care<br>Massou , Efthalia ; University of Cambridge, Public Health and Primary Care<br>Awan, Hassan; Keele University<br>Ford, John; University of Cambridge, Public Health
**Primary Subject Heading**:	Public health
Secondary Subject Heading:	Health policy
Keywords:	STATISTICS & RESEARCH METHODS, Quality in health care < HEALTH SERVICES ADMINISTRATION & MANAGEMENT, Primary Health Care

SCHOLARONE™
Manuscripts

I, the Submitting Author has the right to grant and does grant on behalf of all authors of the Work (as defined in the below author licence), an exclusive licence and/or a non-exclusive licence for contributions from authors who are: i) UK Crown employees; ii) where BMJ has agreed a CC-BY licence shall apply, and/or iii) in accordance with the terms applicable for US Federal Government officers or employees acting as part of their official duties; on a worldwide, perpetual, irrevocable, royalty-free basis to BMJ Publishing Group Ltd ("BMJ") its licensees and where the relevant Journal is co-owned by BMJ to the co-owners of the Journal, to publish the Work in this journal and any other BMJ products and to exploit all rights, as set out in our licence.

The Submitting Author accepts and understands that any supply made under these terms is made by BMJ to the Submitting Author unless you are acting as an employee on behalf of your employer or a postgraduate student of an affiliated institution which is paying any applicable article publishing charge ("APC") for Open Access articles. Where the Submitting Author wishes to make the Work available on an Open Access basis (and intends to pay the relevant APC), the terms of reuse of such Open Access shall be governed by a Creative Commons licence – details of these licences and which Creative Commons licence will apply to this Work are set out in our licence referred to above.

Other than as permitted in any relevant BMJ Author's Self Archiving Policies, I confirm this Work has not been accepted for publication elsewhere, is not being considered for publication elsewhere and does not duplicate material already published. I confirm all authors consent to publication of this Work and authorise the granting of this licence.

For peer review only

Does workforce explain the relationship between funding and patient experience? A mediation analysis of primary care data in England.

Natasha Salant[1]

Efthalia (Lina) Massou[1]

Hassan Awan[2]

John Ford[1]

[1] University of Cambridge

[2] University of Keele

Corresponding author: Natasha Salant, University of Cambridge MPhil Population Health Sciences 2021-2022, natashasalant@gmail.com

For peer review only

ABSTRACT

Objectives: To determine whether GP workforce contributes to the link between practice funding and patient experience. Specifically, to determine whether increased practice funding is associated with better patient experience, and to what degree an increase in workforce accounts for this relationship.

Setting: Primary care practice level analysis of workforce, funding and patient experience of all NHS practices in England.

Primary and secondary outcome measures: The link between NHS-provided funding to general practice (payments per patient) and patient experience, as per the General Practice Patient Survey, was evaluated. Subsequently, mediation analysis, adjusted for covariates, was used to scrutinise the extent to which GP workforce accounts for this relationship (measured as the number of GPs per 10,000 patients).

Participants: We included all general practices in England for which there was relevant data for each primary variable. Atypical practices were excluded, such as those with a patient list size of 0 or where the workforce variable was recorded as being more than 3 SD from the mean. After exclusion, 6139 practices were included in the final analysis.

Results: We found that workforce (GPs per 10,000 population) significantly ($p<0.001$) acts as a mediator in the effect of practice funding on overall patient experience even after adjusting for rurality, sex and age, and deprivation. On average, the mediated effect constitutes 30% of the total effect of practice funding on patient experience.

Conclusions: The increase in the number of doctors in primary care in England appears to be a mechanism through which augmented practice funding could positively impact patient experience. Policy initiatives targeting improved patient experience should prioritise considerations related to workforce and practice funding.

STRENGTHS AND LIMITATIONS

- This study is the first to explore the association between funding, workforce and patient experience through a mediation analysis.
- Given that publicly available datasets are used for this analysis, the data quality of the analysis suffers the same quality issues as these datasets. For instance, there is known inflation of full-time equivalent figures in the NHS Workforce dataset.
- The funding data is limited to NHS payments to practices and therefore does not capture other payments received by practices, for instance for training and teaching.

For peer review only

INTRODUCTION

In recent years, primary care services in England have been subject to increasing demand, tighter budgets, and workforce shortages (1-5). GP workforce has been found to be associated with better self-reported quality of care (6) and both GP and patient satisfaction (7). Workforce inequalities have been identified in previous research with the distribution of general practitioners (GPs), paramedics, and other allied health professionals favouring more affluent areas (7-10)

Patient experience is an important measure of primary health quality because it provides information about the process of care, for instance waiting times and interaction with staff, which cannot be replaced by other indicators (11). Self-reported continuity of care has been linked to cost-effectiveness, reduced emergency hospital admissions, and reduced mortality (12). Historically, patient satisfaction of general practice in the UK has been high; but it has been in decline since 2015 and the gap in patient satisfaction between socio-economic groups have been widening (1,2). On most measures of quality, practices in more deprived areas fare worse: they have lower quality scores, as measured by the Quality Outcomes Framework (QOF), and are more likely to be rated as 'inadequate' by the Care Quality Commission (1).

It has been suggested that funding inequalities are a key driving factor behind inequalities in workforce and satisfaction(1,13). Practices in more deprived areas receive 7% less general practice funding(1). Capitation funding for general practice is positively associated with overall practice quality and Care Quality Commission ratings (14). In addition, there is evidence of an association between funding and patient experience, whereby practices that receive less funding have lower levels of satisfaction(2). However, as it stands, there has been no robust investigation of the mechanisms of this relationship; in other words how lower funding leads to worse patient experience.

Mediation analysis is a statistical method that can be used to evaluate relationships between variables by quantifying the intermediary process through which a predictor variable affects an outcome variable. By using a mediation analysis, we can explore whether the relationship between funding and patient experience could be explained by GP supply. Put simply, we can evaluate whether practices which receive more funding employ more staff, and in turn whether they have better patient reported experience. The impact of socioeconomic deprivation and patient need on the relationships between these variables can also be explored.

For peer review only

METHODS

Data Sources

Four publicly available datasets were used: 'NHS Payments to General Practice' (15), 'General Practice Workforce' (16), GP Patient Survey Data (17), and 'National General Practice Profiles' (18).

Funding data consisted of payments from the NHS to individual general practices in England as part of their contract. Total NHS payments made to general practices included the core capitation amount (global sum) and other payment categories including financial incentives (QOF) and payments for premises. The dataset included total payment and payment per registered and weighted patients (calculated using the Carr-Hill formula (19)).

General Practice workforce data are uploaded by practices every quarter and the database covers all general practices in England. GP data are available for Full Time Equivalent (FTE) across all GP partners, salaried GPs, training grades and locums.

The GP Patient Survey (GPPS) is a national self-reported patient experience survey of over 900,000 adults per year in England (20). It is commissioned by NHS England with the aim of monitoring quality of care. The results are weighted by age, sex, region and socioeconomic status to ensure representativeness (2). Survey results provided practice-level data that is comparable across practices and time (21).

National General Practice Profiles are a set of approximately 150 general practice level indicators that are produced by the Office for Health Improvement and Disparities for practices across England. The Index of Multiple Deprivation (IMD) score is an area-based measure of socio-economic deprivation that comprises of seven distinct weighted domains: Income (22.5%), Employment (22.5%), Health Deprivation and Disability (13.5%), Education, Skills and Training (13.5%), Crime (9.3%), Barriers to Housing and Services (9.3%), and Living Environment (9.3%) (22).

Variables

Predictor

For funding we used payment per registered patient or per weighted patient by dividing 'total NHS payment' by 'registered patient' or 'weighted patient'.

Outcome

We used four patient experience variables from the GP Patient Survey based on previous studies(2,13).

(a) proportion of respondents who had a good or very good experience of making an appointment (Access),
(b) proportion of respondents who always see their preferred GP (Continuity),
(c) proportion of respondents who had trust and confidence in their GP (Trust), and
(d) proportion of respondents who had an overall good or very good experience (Overall Experience).

Mediator

We used full time equivalent (FTE) GPs per 10,000 registered or weighted patients as the mediator.

For peer review only

Covariates

An adjusted model was undertaken using patient weights and covariates. Weighted payments per patient takes into account patient sex, age, long-standing health condition and rurality, so these were not added as separate variables. IMD was also added as a covariate.

Sample

As illustrated in Figure 1Sup, we included all general practices in England with data available. Where practices were entirely missing from payments, workforce and patient experience datasets, they were excluded. In addition, atypical practices were excluded, for example if the patient list size was 0 or where workforce data were improbable (defined as more the 3 standard deviations from the mean).

Data merge

Relevant variables from data sources were linked using practice code, and sense-checked by scanning that practice names matched. To account for the lag between funding, employment and patient experience, 2017/18 funding data were used, December 2018 workforce data and March 2019 patient experience data. Pandemic data were not used because of data quality issues and challenges around accounting for the impact of changes to services.

Statistical analysis

Three mediation model structures were built using the Baron and Kenny approach (23) also known as the causal steps approach, and all models assumed the causal structure in Figures 1 and 2. These were: an unadjusted model (where GPs per registered patient was tested as a mediator of the effect of payments per registered patient on patient experience), a weighted patients' model (where GPs per weighted patient was tested as a mediator of payments per weighted patient on patient experience) and a deprivation adjusted model (where the unadjusted model was adjusted for deprivation). The weighted model was weighted using the Carr-Hill formula which takes into account, patient sex, age, long-standing health condition and rurality. For each model structure, there were four distinct models that tested each distinct patient experience variable.

In total, twelve mediation models were tested. For each, a simple linear regression of the predictor on the outcome was run to test whether there was a significant association. Mediation analysis was considered if the outcome variable was significantly associated with the predictor variable, the mediator variable was significantly associated with the predictor variable, and controlling for the predictor variable, that the mediator variable associated with the outcome variable. The significance of indirect effect was tested using bootstrapping procedures. Unstandardised indirect effects were computed for each of 1000 bootstrapped samples, and the 95% confidence interval was computed by determining the indirect effects at the 2.5th and 97.5th percentiles. If mediation was established using simple linear regressions, the model was adjusted for patient sex, age, long-standing health condition and rurality (24,25).

In addition, the model's sensitivity to removing outliers was tested by running the same analyses on datasets that did not remove outliers.

All analyses were conducted using R (version 4.2) and RStudio.

For peer review only

Patient and Public Involvement

No patient involved.

For peer review only

RESULTS

The final number of practices included ranged from 5,946 to 6,139 (Table 1) out of a total of 6836 practices as at 2019. Our results therefore account for approximately 90% of all practices in England. The continuity dataset included the fewest practices because the survey's continuity question was answered by 2% fewer participants than access, trust and overall experience questions. Across all datasets, the average practice receives NHS payments of £152 per registered patient and the average practice size is 8,640 patients. Most practices have a General Medical Services (GMS) contract, do not dispense and are based in urban areas (Table 1Sup presents extended practice characteristics). Practices employ on average 5.6 FTE GPs. The average practice receives a good or very good rating of 83% for overall patient experience, 69% for access, 49% for continuity and 95% for trust.

Table 1: Practice characteristics for each outcome dataset

	Access	Continuity	Trust	Overall Experience
	Total (N=6137)	Total (N=5946)	Total (N=6137)	Total (N=6139)
Payments per Patient (£) Mean (SD)	152 (40.3)	152 (39.4)	152 (40.3)	152 (40.3)
Payments per Weighted Patient (£) Mean (SD)	151 (35.4)	151 (34.7)	151 (35.4)	151 (35.4)
Rurality n (%)				
Rural	919 (15.0%)	885 (14.9%)	919 (15.0%)	919 (15.0%)
Urban	5218 (85.0%)	5061 (85.1%)	5218 (85.0%)	5220 (85.0%)
Registered Patients Mean (SD)	8640 (5220)	8790 (5150)	8640 (5220)	8640 (5220)
FTE GPs per 10,000 patients (pts) Mean (SD)	5.61 (2.20)	5.62 (2.19)	5.61 (2.20)	5.61 (2.20)
Total Patient number > 65 yrs Mean (SD)	1620 (1210)	1650 (1200)	1620 (1210)	1620 (1210)
% Patients with long-standing health condition Mean (SD)	51.3 (8.58)	51.3 (8.58)	51.3 (8.58)	51.3 (8.59)
Patient Experience rated good (%) Mean (SD)	69.1 (14.4)	49.1 (18.5)	95.3 (3.80)	83.4 (9.74)
[Min, Max]	[19.1, 100]	[2.14, 98.0]	[71.6, 100]	[32.2, 100]
Response Rate (%)				

For peer review only

Mean (SD)	35.9 (10.8)	36.0 (10.8)	35.9 (10.8)	35.8 (10.8)

<u>*Does workforce mediate the relationship between funding and overall patient outcomes?*</u>

Unadjusted analysis

In the unadjusted model, the effect of funding on overall patient experience was partially mediated via GPs per 10,000 patients (Figure 3). The proportion of the relationship between funding and overall patient experience that is mediated by GPs per 10,000 is 32%. In other words, the increase in payments per patient that drives an increase in GPs per 10,000 patients explains more than one third of the effect on an increase in overall patient experience.

The regression coefficient between funding and overall patient experience was statistically significant, as was the regression coefficient between GPs per 10,000 patients and overall patient experience (p<0.001). The total effect of funding on overall patient experience was 0.044. The indirect effect via workforce was 0.014 (95%CI 0.011 to 0.017). The model could be seen to illustrate that a £10 increase in practice funding is associated with a 0.44% increase in overall patient experience.

Analysis adjusted for socio-economic deprivation

When adjusted for socio-economic deprivation, the effect of funding on overall patient experience is still significantly (p<0.001) partially mediated by GPs per 10,000 patients (Figure 4). In this adjusted model, the indirect effect via GP workforce was 0.013 (95% CI, 0.011 to 0.015). The total effect of funding on overall patient experience (0.043) has the same effect size as the unadjusted model and is still significant (p<0.001). However, the direct effect (0.030) and indirect effect (0.013), while still both significant (p<0.001), differ slightly in size from the unadjusted model, and the proportion mediated is 30%.

Analysis using weighted funding data

Figure 5 shows the effect of funding on overall patient experience was partially mediated via GPs per 10,000 weighted patients. The indirect effect via workforce was 0.010 (95%CI 0.008 to 0.012). This weighted model could be seen to illustrate that a £10 increase in practice funding is associated with a 0.38% increase in overall patient experience, and the proportion of the total effect that weighted funding has on overall patient experience is 26%.

The relationship between weighted funding and overall patient experience (0.028) was significant and similar to the unadjusted model, as was the relationship between GPs per 10,000 patients and overall patient experience (1.152).

<u>*Does workforce mediate the relationship between funding and access, trust and continuity?*</u>

Unadjusted analysis

Mediation models with access, trust and continuity indicators as outcome variables were all similarly significantly mediated (p<0.001) by GPs per 10,000 patients (Table 2Sup). The 'access' outcome had the strongest association between funding and patient experience, with the unadjusted model demonstrating that a £10 pound increase in practice funding was associated with a 0.44% increase in patient experience, of which 36% could be explained by an increase of 1 GP per 10,000 patients.

Adjusted analysis

For peer review only

When using weighted data and adjusting for deprivation, the ‘access’ effect size remained the highest out of all models, with GP workforce accounting for ~31% of the effect size. Direct, total and mediated effect sizes were substantially smaller for the continuity and trust models indicating that while an increase in GPs per 10,000 patients was a statistically significant mediator for trust and continuity measures of patient experience, the effect may be more relevant for access and overall experience measures.

Sensitivity analyses including the data outliers did not have a substantial impact on the results.

For peer review only

Main finding of this study

We found that the number of GPs per 10,000 patients significantly ($p<0.001$) mediated the effect of practice funding on overall patient experience, access, trust and continuity. In simpler terms, practices with more funding had better patient experience and some of this relationship can be accounted for by an increase in workforce. However, it is crucial to note that the effect sizes are relatively modest. In the unadjusted model, the total effect size of the association between payments per patient and overall patient experience is 0.044. This implies that a £10 increase in payments per patient is linked to a 0.4% rise in overall patient experience. Considering the mean payment per patient is £152, an average practice would experience only a marginal increase in overall patient experience, even with a funding boost of 6%. Nonetheless, our findings indicate that 30% of the explanation for the link between an increase in payments per patient and improved overall patient experience is attributed to an increase in FTE GPs per 10,000 patients.

Strengths and limitations

The study was strengthened by building up the mediation model step by step, allowing an understanding of the associations at each level of the mediation. In addition, we used a non-parametric estimation of the indirect effect and significance, which, because of its wide applicability to a variety of models, resolves any doubts arising from whether or not the method of estimation and significance testing is suitable for this model (24, 25).

GP Patient Survey response rate is about 30%, raising questions about whether the data is suitable for a quantitative England-wide study. However, because responses are weighted to account for selection bias, demographic characteristics of the eligible population, as well as differences between responders and non-responders (26), this problem is mitigated. In addition, we have only identified four patient experience outcomes for this study, meaning there may be other components which had different results. For example, waiting times may have a stronger relationship with workforce and funding.

Both the General Practice Workforce and NHS Payments datasets have known quality considerations. For the Workforce dataset, there is known inflation of FTE and headcount figures due to the recruitment of temporary staff to cover staff on long-term leave (27). For the NHS Payments dataset, patient list numbers should be viewed with caution given that there is known list inflation whereby patients who have left a practice are not deregistered (28).

This analysis of practice funding is limited to an analysis of NHS payments to practice and does not include practice funding received from other sources such as research or training. However, this limitation addresses a wider problem that practice-level data on additional funding, expenditure and profit data is not publicly available.

What this study adds

This study is the first to explore the association between practice-level NHS payments per patient, patient experience and workforce through mediation analyses. Despite prior evidence to show that an increase in NHS practice funding is associated with an increase in patient experience (2) and CQC scores (12), it is unclear from these studies how increasing practice funding may lead to improved quality outcomes. Understanding the mechanisms of action is valuable to recognise correlates of increasing GP per patient ratios, identify practices that may be more or less likely to benefit from an

For peer review only

increase in payments per patient, or inform policy so that increasing practice funding can be more strategic. Payments per weighted patient are not equal per decile, with more affluent deciles receiving more funding per weighted patient. That information is enriched by the finding that the inequality in payments is associated with an inequality in capability to recruit GPs, which plays a substantial role in patient experience.

Moreover, this study read together with previous analyses on the widening inequality GP distribution (9, 10) should be interpreted to mean that if the status quo continues with regard to NHS payments to practices, i.e. a total increase in average payments per patient of approximately 4% over the past four years (29), the inequality in payments per weighted patient, as well as that in GPs per 10,000 weighted patients, will likely continue to grow. Given the results of this study, it is unsurprising that recent results from the GP Patient Survey reveal a widening gap in patient experience, with patients attending practices in more deprived neighbourhoods reporting worse overall experience than patients attending practices in more affluent neighbourhoods (30).

Investment in general practice in England is thought to have lagged behind what is necessary to finance increasing total expenditure, population growth, increasing consultations and expanding complexity of health needs (29). While undeniable value for money can be achieved with primary care investment (31, 32), the results of this study do not suggest that simply investing more money in practices would solve the problem. While practice payments per patient significantly impact the number of GPs per 10,000 patients, the effect size is small. This reflects that workforce recruitment is a problem that is not entirely dependent on practice funding. However, if the system is committed to improving quality, policy must target both practice funding, GP recruitment and quality improvement.

Policy recommendations

The contractual funding model (Carr-Hill formula) should be reviewed to rectify the unequal distribution of payments per weighted patient. Its inaccurate reflection of patient need becomes apparentin the inequality of average practice payments per weighted patients. As discerned in our analysis this has an impact on the distribution of GPs per 10,000 patients as well as on inequality of patient experience. In addition, given the effect of practice funding on GPs per 10,000 patients identified by this study, as well as the widening inequality of GP distribution found in multiple previous studies, it is recommended that additional funding is identified and targeted towards underdoctored areas to stop the inequality from increasing. This targeted investment could have a knock-on effect on patient experience in these areas, according to the findings of this study. Finally, given the demonstrable link between funding, GP supply and patient experience, it is recommended that practices achieving low quality of care scores are not penalised by withholding financial incentives. Instead, a supportive approach should be adopted, fostering improvement in the quality of care provided by these practices.

The formulation and implementation of these recommended policy reforms require inclusive input from a diverse cadre of primary care practitioners, as well as the active inclusion of a robust patient perspective.

For peer review only

CONCLUSION

We found that the number of GPs per 10,000 patients is a significant mediator and explains a third of the relationship between funding and patient experience. However the effect is small, meaning that a substantial increase in funding would be required to meaningfully improve patient experience. Funding increases should therefore be implemented alongside policy reform to improve patient experience and reduce inequalities.

Figure Legend

Figure 1: Assumed causal structure of unadjusted mediation model

Figure 2: Assumed causal structure of adjusted mediation model

Figure 3: Unadjusted mediation model results

Figure 4: Deprivation adjusted mediation model results

Figure 5: Carr-Hill adjusted mediation model results

Supplemental Material

Figure 1Sup: Data sample flow chart

Table 1Sup: Extended practice characteristics for each outcome dataset

Table 2Sup: Access, Continuity and Trust mediation models

Contributorship statement

Natasha Salant is the primary author having made substantial contributions to the conception and design of the work, the acquisition of the data, the cleaning and analysis of the data as well as the interpretation thereof. She is also responsible for drafting of the work, revising it, and final approval for it to be published, thereby agreeing to be accountable for all aspects of the work. Hassan Awan made substantial contributions to the interpretation of data, the critical revision and final approval for publication thereby agreeing to be accountable for the work. Efthalia Massou made substantial contributions to the design of the statistical analysis as well as the interpretation of the analysis, critical revision of the draft, and final approval for publication thereby being accountable for the work. John Ford conceptualised the initial idea and made substantial contributions to the concept and design of the work as well as to the interpretation of the data, critical revision of the draft, and final approval for publication thereby agreeing to be accountable for the work.

For peer review only

Competing Interests

No authors have any conflicting interests to declare.

Funding

Natasha Salant is grateful to Chevening Fellowships, the UK government's global scholarship programme, funded by the Foreign, Commonwealth and Development Office (FCDO) and partner organisations for the funding of her MPhil program, thereby making this research possible.

Data sharing statement

Datasets used are available publicly as per references provided for each dataset used. Code for analysis is available upon request.

Ethics Approval

This study does not involve human participants and ethical approval was not required.

For peer review only

References

1. Level or not? - The Health Foundation [Internet]. [cited 2022 Jan 1]. Available from: https://www.health.org.uk/publications/reports/level-or-not

2. L'Esperance V, Gravelle H, Schofield P, Ashworth M. Impact of primary care funding on patient satisfaction: a retrospective longitudinal study of English general practice, 2013–2016. Br J Gen Pract. 2020 Dec 1;71(702):e47–54.

3. The bigger picture - The Health Foundation [Internet]. [cited 2022 Jul 16]. Available from: https://www.health.org.uk/publications/reports/the-bigger-picture

4. Tackling the inverse care law - The Health Foundation [Internet]. [cited 2022 Jul 8]. Available from: https://www.health.org.uk/publications/reports/tackling-the-inverse-care-law

5. 'Levelling up' general practice in England - The Health Foundation [Internet]. [cited 2022 Jun 24]. Available from: https://www.health.org.uk/publications/long-reads/levelling-up-general-practice-in-england

6. Vallejo-Torres L, Morris S. Primary care supply and quality of care in England. Eur J Health Econ. 2018;19(4):499–519.

7. Gibson J, Francetic I, Spooner S, Checkland K, Sutton M. Primary care workforce composition and population, professional, and system outcomes: a retrospective cross-sectional analysis. Br J Gen Pract. 2022 May 1;72(718):e307–15.

8. Cookson R, Propper C, Asaria M, Raine R. Socio-Economic Inequalities in Health Care in England. Fisc Stud. 2016;37(3–4):371–403.

9. Nussbaum C, Massou E, Fisher R, Morciano M, Harmer R, Ford J. Inequalities in the distribution of the general practice workforce in England: a practice-level longitudinal analysis. BJGP Open [Internet]. 2021 Oct 1 [cited 2022 Apr 12];5(5). Available from: https://bjgpopen.org/content/5/5/BJGPO.2021.0066

10. Asaria M, Cookson R, Fleetcroft R, Ali S. Unequal socioeconomic distribution of the primary care workforce: whole-population small area longitudinal study. BMJ Open. 2016 Jan 19;6(1):e008783.

11. Schoenfelder T. Patient Satisfaction: A Valid Indicator for the Quality of Primary Care? Prim Health Care Open Access [Internet]. 2012 [cited 2022 Mar 23];02(04). Available from: https://www.omicsonline.org/open-access/patient-satisfaction-a-valid-indicator-for-the-quality-of-primary-care-2167-1079.1000e106.php?aid=8310

12. Pereira Gray DJ, Sidaway-Lee K, White E, Thorne A, Evans PH. Continuity of care with doctors—a matter of life and death? A systematic review of continuity of care and mortality. BMJ Open. 2018 Jun;8(6):e021161.

13. Saunders CL, Flynn S, Massou E, Lyratzopoulos G, Abel G, Burt J. Sociodemographic inequalities in patients' experiences of primary care: an analysis of the General Practice Patient Survey in England between 2011 and 2017. J Health Serv Res Policy. 2021 Jul 1;26(3):198–207.

For peer review only

14. L'Esperance V, Gravelle H, Schofield P, Santos R, Ashworth M. Relationship between general practice capitation funding and the quality of primary care in England: a cross-sectional, 3-year study. BMJ Open. 2019 Nov 7;9(11):e030624.

15. NHS Payments to General Practice - NHS Digital [Internet]. [cited 2022 Jan 31]. Available from: https://digital.nhs.uk/data-and-information/publications/statistical/nhs-payments-to-general-practice

16. General Practice Workforce [Internet]. NHS Digital. [cited 2022 Jan 31]. Available from: https://digital.nhs.uk/data-and-information/publications/statistical/general-and-personal-medical-services

17. Survey and Reports [Internet]. [cited 2022 Feb 3]. Available from: https://www.gp-patient.co.uk/surveysandreports

18. National General Practice Profiles - OHID [Internet]. [cited 2022 Feb 5]. Available from: https://fingertips.phe.org.uk/profile/general-practice

19. Dyson M. Global sum allocation formula [Internet]. The British Medical Association is the trade union and professional body for doctors in the UK. [cited 2021 Dec 8]. Available from: https://www.bma.org.uk/advice-and-support/gp-practices/funding-and-contracts/global-sum-allocation-formula

20. GP Patient Survey – About [Internet]. [cited 2022 Feb 3]. Available from: https://www.gp-patient.co.uk/About

21. Tallett A, Poots AJ, Graham C, Peters M, Corbett R, Sizmur S, et al. How can patient experience scores be used to predict quality inspection ratings? A retrospective cross-sectional study of national primary care datasets in the UK. BMJ Open. 2020 Nov 26;10(11):e041709.

22. English indices of deprivation [Internet]. GOV.UK. [cited 2022 Feb 9]. Available from: https://www.gov.uk/government/collections/english-indices-of-deprivation

23. Baron RM, Kenny DA. The moderator–mediator variable distinction in social psychological research: Conceptual, strategic, and statistical considerations. J Pers Soc Psychol. 1986 Dec;51(6):1173–82.

24. Imai K, Keele L, Yamamoto T. Identification, Inference and Sensitivity Analysis for Causal Mediation Effects. Stat Sci [Internet]. 2010 Feb 1 [cited 2022 Jul 13];25(1). Available from: https://projecteuclid.org/journals/statistical-science/volume-25/issue-1/Identification-Inference-and-Sensitivity-Analysis-for-Causal-Mediation-Effects/10.1214/10-STS321.full

25. Imai K, Keele L, Tingley D. A general approach to causal mediation analysis. Psychol Methods. 2010 Dec;15(4):309–34.

26. GPPS Technical Annex 2019 [Internet]. [cited 2022 Jul 14]. Available from: https://www.gp-patient.co.uk/downloads/2019/GPPS_2019_Technical_Annex_PUBLIC.pdf

27. Background Data Quality Statement [Internet]. NHS Digital. [cited 2022 Jul 15]. Available from: https://digital.nhs.uk/data-and-information/publications/statistical/general-and-personal-medical-services/31-october-2021/background-data-quality-statement

For peer review only

28. Executive Summary [Internet]. NHS Digital. [cited 2022 Jul 15]. Available from: https://digital.nhs.uk/data-and-information/publications/statistical/nhs-payments-to-general-practice/england-2020-21/executive-summary-copy

29. Dyson M. Investment in general practice [Internet]. The British Medical Association is the trade union and professional body for doctors in the UK. [cited 2022 Jul 27]. Available from: https://www.bma.org.uk/advice-and-support/nhs-delivery-and-workforce/funding/investment-in-general-practice

30. Statistics » GP Patient Survey 2022 [Internet]. [cited 2022 Jul 15]. Available from: https://www.england.nhs.uk/statistics/2022/07/14/gp-patient-survey-2022/#

31. Starfield B, Shi L, Macinko J. Contribution of Primary Care to Health Systems and Health. Milbank Q. 2005;83(3):457–502.

32. Schäfer WLA, Boerma WGW, van den Berg MJ, De Maeseneer J, De Rosis S, Detollenaere J, et al. Are people's health care needs better met when primary care is strong? A synthesis of the results of the QUALICOPC study in 34 countries. Prim Health Care Res Dev. 2019 Jul 1;20:e104.

For peer review only

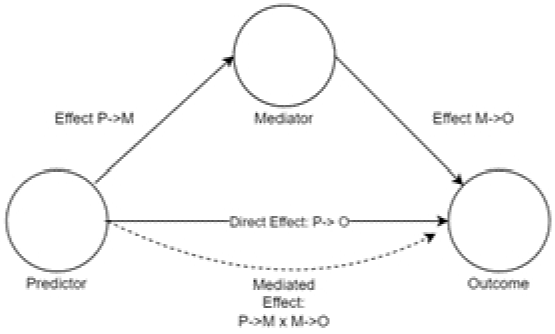

Figure 1

191x118mm (38 x 38 DPI)

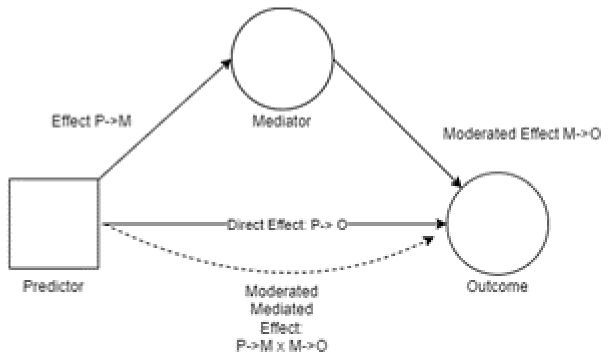

Figure 2

207x118mm (38 x 38 DPI)

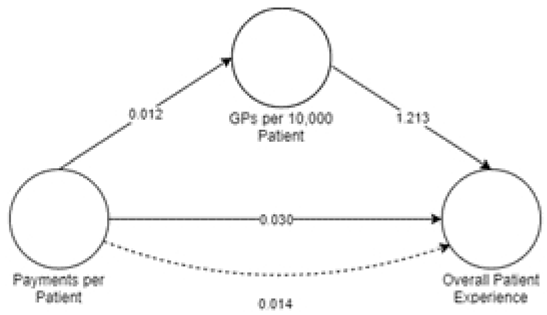

Figure 3

181x101mm (38 x 38 DPI)

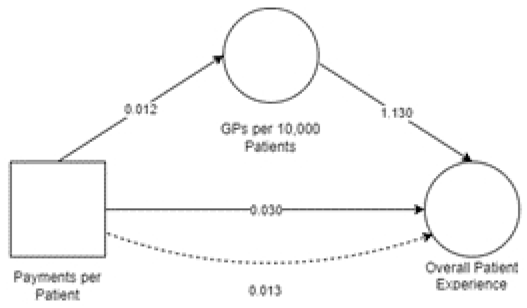

Figure 4

174x102mm (38 x 38 DPI)

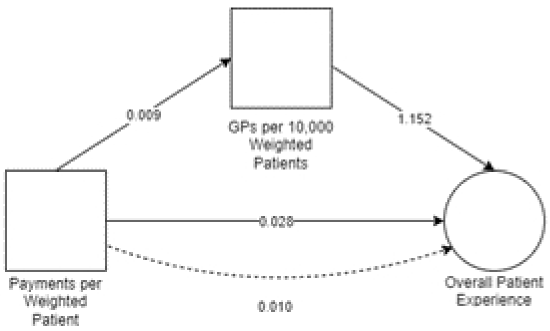

Figure 5

184x108mm (38 x 38 DPI)

Figure 1Sup: Data sample flow chart

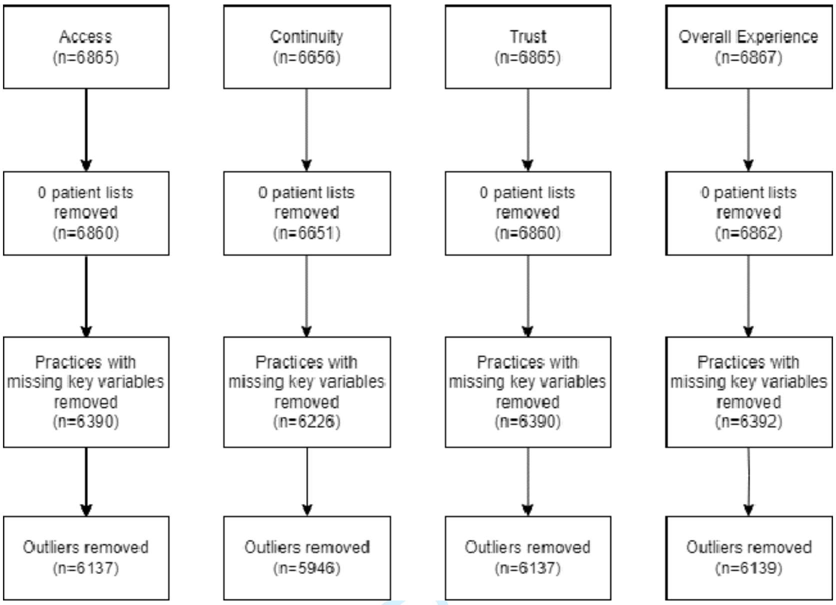

Table 1Sup: Extended practice characteristics for each outcome dataset

	Access	*Continuity*	*Trust*	*Overall Experience*
	Total (N=6137)	Total (N=5946)	Total (N=6137)	Total (N=6139)
Total NHS Payments (£) *Mean (SD)*	1,300,000 (806,000)	1,320,000 (794,000)	1,300,000 (806,000)	1,300,000 (806,000)
Payments per Patient (£) *Mean (SD)*	152 (40.3)	152 (39.4)	152 (40.3)	152 (40.3)
Payments per Weighted Patient (£) *Mean (SD)*	151 (35.4)	151 (34.7)	151 (35.4)	151 (35.4)
Contract Type n (%) *APMS*	136 (2.2%)	125 (2.1%)	136 (2.2%)	136 (2.2%)

For peer review only

GMS	4313 (70.3%)	4176 (70.2%)	4313 (70.3%)	4315 (70.3%)
PMS	1688 (27.5%)	1645 (27.7%)	1688 (27.5%)	1688 (27.5%)
Unknown	0 (0%)	0 (0%)	0 (0%)	0 (0%)
Dispensing n (%)				
Yes	860 (14.0%)	833 (14.0%)	860 (14.0%)	860 (14.0%)
No	5277 (86.0%)	5113 (86.0%)	5277 (86.0%)	5279 (86.0%)
Unknown	0 (0%)	0 (0%)	0 (0%)	0 (0%)
Rurality n (%)				
Rural	919 (15.0%)	885 (14.9%)	919 (15.0%)	919 (15.0%)
Urban	5218 (85.0%)	5061 (85.1%)	5218 (85.0%)	5220 (85.0%)
Registered Patients				
Mean (SD)	8640 (5220)	8790 (5150)	8640 (5220)	8640 (5220)
Weighted Patients				
Mean (SD)	8630 (5120)	8780 (5060)	8630 (5120)	8630 (5120)
FTE GPs per 10,000 patients (pts)				
Mean (SD)	5.61 (2.20)	5.62 (2.19)	5.61 (2.20)	5.61 (2.20)
Total Patient number > 65 yrs				
Mean (SD)	1620 (1210)	1650 (1200)	1620 (1210)	1620 (1210)
% Patients with long-standing health condition				
Mean (SD)	51.3 (8.58)	51.3 (8.58)	51.3 (8.58)	51.3 (8.59)

For peer review only

Patient Experience rating (%)<br>*Mean (SD)*<br>*[Min, Max]*	69.1 (14.4)<br>[19.1, 100]	49.1 (18.5)<br>[2.14, 98.0]	95.3 (3.80)<br>[71.6, 100]	83.4 (9.74)<br>[32.2, 100]
Response Rate (%)<br>*Mean (SD)*	35.9 (10.8)	36.0 (10.8)	35.9 (10.8)	35.8 (10.8)

Table 2Sup: Access, Continuity and Trust mediation models

	Access		Continuity		Trust	
	Coefficient	CI: 95%	Coefficient	CI: 95%	Coefficient	CI: 95%
Unadjusted Model						
**Direct Effect of predictor on outcome**	0.044	(0.037; 0.06)	0.044	(0.030; 0.06)	0.007	(0.006; 0.01)
**Total Effect of predictor on outcome**	0.062	(0.053; 0.07)	0.050	(0.036; 0.06)	0.013	(0.010; 0.01)
**Indirect (Mediated) Effect**	0.016	(0.013; 0.02)	0.006	(0.004; 0.01)	0.005	(0.004; 0.01)
Carr-Hill adjusted Model						
**Direct Effect of predictor on outcome**	0.048	(0.037; 0.06)	0.037	(0.022; 0.06)	0.007	(0.004; 0.01)

For peer review only

**Total Effect of predictor on outcome**	0.060	(0.048; 0.07)	0.041	(0.026; 0.06)	0.010	(0.0073; 0.01)
**Indirect (Mediated) Effect**	0.011	(0.009; 0.01)	0.004	(0.002; 0.01)	0.003	(0.0025; 0.0035)
Deprivation adjusted Model<br>**Direct Effect of predictor on outcome**	0.047	(0.038; 0.06)	0.045	(0.030; 0.06)	0.008	(0.006; 0.010)
**Total Effect of predictor on outcome**	0.061	(0.053; 0.07)	0.050	(0.035; 0.06)	0.012	(0.010; 0.014)
**Indirect (Mediated) Effect**	0.015	(0.012; 0.02)	0.005	(0.003; 0.01)	0.004	(0.003; 0.005)

For peer review only

The RECORD statement – checklist of items, extended from the STROBE statement, that should be reported in observational studies using routinely collected health data.

	Item No.	STROBE items	Location in manuscript where items are reported	RECORD items	Location in manuscript where items are reported
**Title and abstract**					
	1	(a) Indicate the study's design with a commonly used term in the title or the abstract (b) Provide in the abstract an informative and balanced summary of what was done and what was found		RECORD 1.1: The type of data used should be specified in the title or abstract. When possible, the name of the databases used should be included.<br><br>RECORD 1.2: If applicable, the geographic region and timeframe within which the study took place should be reported in the title or abstract.<br><br>RECORD 1.3: If linkage between databases was conducted for the study, this should be clearly stated in the title or abstract.	Methods<br><br>pg 4
**Introduction**					
Background rationale	2	Explain the scientific background and rationale for the investigation being reported			
Objectives	3	State specific objectives, including any prespecified hypotheses			
**Methods**					
Study Design	4	Present key elements of study design early in the paper			
Setting	5	Describe the setting, locations, and relevant dates, including periods of recruitment, exposure, follow-up, and data collection			

For peer review only

Participants	6	*(a) Cohort study* - Give the eligibility criteria, and the sources and methods of selection of participants. Describe methods of follow-up<br>*Case-control study* - Give the eligibility criteria, and the sources and methods of case ascertainment and control selection. Give the rationale for the choice of cases and controls<br>*Cross-sectional study* - Give the eligibility criteria, and the sources and methods of selection of participants<br><br>*(b) Cohort study* - For matched studies, give matching criteria and number of exposed and unexposed<br>*Case-control study* - For matched studies, give matching criteria and the number of controls per case		RECORD 6.1: The methods of study population selection (such as codes or algorithms used to identify subjects) should be listed in detail. If this is not possible, an explanation should be provided.<br><br>RECORD 6.2: Any validation studies of the codes or algorithms used to select the population should be referenced. If validation was conducted for this study and not published elsewhere, detailed methods and results should be provided.<br><br>RECORD 6.3: If the study involved linkage of databases, consider use of a flow diagram or other graphical display to demonstrate the data linkage process, including the number of individuals with linked data at each stage.	Methods<br><br>pg 4
Variables	7	Clearly define all outcomes, exposures, predictors, potential confounders, and effect modifiers. Give diagnostic criteria, if applicable.		RECORD 7.1: A complete list of codes and algorithms used to classify exposures, outcomes, confounders, and effect modifiers should be provided. If these cannot be reported, an explanation should be provided.	Methods<br><br>pg 4 and 5
Data sources/ measurement	8	For each variable of interest, give sources of data and details of methods of assessment (measurement).<br>Describe comparability of assessment methods if there is more than one group			

For peer review only

Bias	9	Describe any efforts to address potential sources of bias			
Study size	10	Explain how the study size was arrived at			
Quantitative variables	11	Explain how quantitative variables were handled in the analyses. If applicable, describe which groupings were chosen, and why			
Statistical methods	12	(a) Describe all statistical methods, including those used to control for confounding<br>(b) Describe any methods used to examine subgroups and interactions<br>(c) Explain how missing data were addressed<br>(d) *Cohort study* - If applicable, explain how loss to follow-up was addressed<br>*Case-control study* - If applicable, explain how matching of cases and controls was addressed<br>*Cross-sectional study* - If applicable, describe analytical methods taking account of sampling strategy<br>(e) Describe any sensitivity analyses			
Data access and cleaning methods		..		RECORD 12.1: Authors should describe the extent to which the investigators had access to the database population used to create the study population.	Methods<br>pg 4

For peer review only

				RECORD 12.2: Authors should provide information on the data cleaning methods used in the study.	
Linkage		..		RECORD 12.3: State whether the study included person-level, institutional-level, or other data linkage across two or more databases. The methods of linkage and methods of linkage quality evaluation should be provided.	Methods<br>pg 4
**Results**					
Participants	13	(a) Report the numbers of individuals at each stage of the study (*e.g.*, numbers potentially eligible, examined for eligibility, confirmed eligible, included in the study, completing follow-up, and analysed)<br>(b) Give reasons for non-participation at each stage.<br>(c) Consider use of a flow diagram		RECORD 13.1: Describe in detail the selection of the persons included in the study (*i.e.*, study population selection) including filtering based on data quality, data availability and linkage. The selection of included persons can be described in the text and/or by means of the study flow diagram.	Methods<br>Abstract pg 1
Descriptive data	14	(a) Give characteristics of study participants (*e.g.*, demographic, clinical, social) and information on exposures and potential confounders<br>(b) Indicate the number of participants with missing data for each variable of interest<br>(c) *Cohort study* - summarise follow-up time (*e.g.*, average and total amount)			
Outcome data	15	*Cohort study* - Report numbers of outcome events or summary measures over time<br>*Case-control study* - Report numbers in each exposure			

For peer review only

		category, or summary measures of exposure *Cross-sectional study* - Report numbers of outcome events or summary measures			
Main results	16	(a) Give unadjusted estimates and, if applicable, confounder-adjusted estimates and their precision (e.g., 95% confidence interval). Make clear which confounders were adjusted for and why they were included (b) Report category boundaries when continuous variables were categorized (c) If relevant, consider translating estimates of relative risk into absolute risk for a meaningful time period			
Other analyses	17	Report other analyses done—e.g., analyses of subgroups and interactions, and sensitivity analyses			
**Discussion**					
Key results	18	Summarise key results with reference to study objectives			
Limitations	19	Discuss limitations of the study, taking into account sources of potential bias or imprecision. Discuss both direction and magnitude of any potential bias		RECORD 19.1: Discuss the implications of using data that were not created or collected to answer the specific research question(s). Include discussion of misclassification bias, unmeasured confounding, missing data, and changing eligibility over time, as they pertain to the study being reported.	Discussion pg 10
Interpretation	20	Give a cautious overall interpretation of results considering objectives,			

For peer review only

		limitations, multiplicity of analyses, results from similar studies, and other relevant evidence			
Generalisability	21	Discuss the generalisability (external validity) of the study results			
**Other Information**					
Funding	22	Give the source of funding and the role of the funders for the present study and, if applicable, for the original study on which the present article is based			
Accessibility of protocol, raw data, and programming code		..		RECORD 22.1: Authors should provide information on how to access any supplemental information such as the study protocol, raw data, or programming code.	pg 13

*Reference: Benchimol EI, Smeeth L, Guttmann A, Harron K, Moher D, Petersen I, Sørensen HT, von Elm E, Langan SM, the RECORD Working Committee. The REporting of studies Conducted using Observational Routinely-collected health Data (RECORD) Statement. *PLoS Medicine* 2015; in press.

*Checklist is protected under Creative Commons Attribution (CC BY) license.

For peer review only
